# Ivermectin represses Wnt/β-catenin signaling by binding to TELO2, a regulator of phosphatidylinositol 3-kinase-related kinases

**DOI:** 10.1016/j.isci.2022.103912

**Published:** 2022-03-07

**Authors:** Honami Yonezawa, Akari Ikeda, Ryo Takahashi, Haruka Endo, Yasuyo Sugawara, Mikako Goto, Mirute Kanno, Sosuke Ogawa, Karin Nakamura, Haruki Ujiie, Masato Iwatsuki, Tomoyasu Hirose, Toshiaki Sunazuka, Yoshimasa Uehara, Naoyuki Nishiya

**Affiliations:** 1 Division of Integrated Information for Pharmaceutical Sciences, Department of Clinical Pharmacy, Iwate Medical University School of Pharmacy, Shiwa-gun, Yahaba-cho, Iwate 028-3694, Japan; 2 Ōmura Satoshi Memorial Institute and Graduate School of Infection Control Sciences, Kitasato University, Tokyo, Minato-ku 108-8641, Japan

**Keywords:** Biochemistry, Small molecule, Molecular biology

## Abstract

Ivermectin (IVM), an avermectin-derivative anthelmintic, specifically binds to glutamate-gated chloride ion channels (GluCls), causing paralysis in invertebrates. IVM also exhibits other biological activities such as Wnt/β-catenin pathway inhibition in vertebrates that do not possess GluCls. This study showed that affinity purification using immobilized IVM B1a isolated TELO2, a cofactor of phosphatidylinositol 3-kinase-related kinases (PIKKs), as a specific IVM B1a-binding protein. *TELO2* knockdown reduced cytoplasmic β-catenin and the transcriptional activation of β-catenin/TCF. IVM B1a bound to TELO2 through the C-terminal α-helix, in which mutations conferred IVM resistance. IVM reduced the TELO2 and PIKK protein levels and the AKT and S6 kinase phosphorylation levels. The inhibition of mTOR kinase reduced the cytoplasmic β-catenin level. Therefore, IVM binds to TELO2, inhibiting PIKKs and reducing the cytoplasmic β-catenin level. In conclusion, our data indicate TELO2 as a druggable target for human diseases involving abnormalities of the Wnt/β-catenin pathway and PIKKs, including mTOR.

## Highlights

• Ivermectin is a chemical suppressor of the eyeless phenotype in zebrafish embryos
• Ivermectin physically interacts with TELO2
• TELO2 mediates Wnt/β-catenin signaling inhibition by ivermectin
• Ivermectin reduces the PIKK protein levels and downstream signaling

## Graphical abstract

**Figure:**
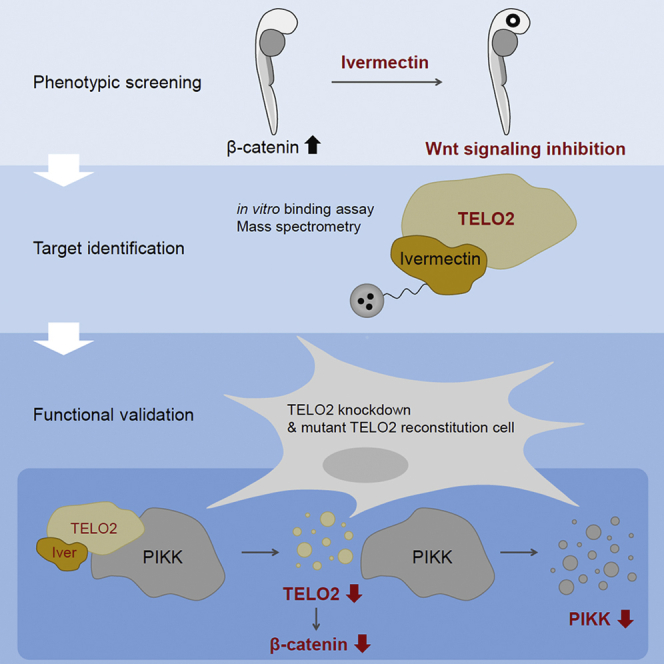

## Introduction

Natural compounds are diverse in structure and biological activities. Macrocyclic structures, often found in bioactive compounds, have been recognized as new modalities that exert a mode of action distinct from that of the conventional small molecules (Driggers et al., 2008). Cyclosporin A, tacrolimus, and rapamycin exemplify bivalent compounds that bind to two proteins simultaneously (Sabatini et al., 1994; Sabers et al., 1995; Schreiber and Crabtree, 1992). Macrocycles bind to low-druggability targets with increased contact area compared with conventional small molecules (Villar et al., 2014). Thus, macrocycles have garnered scientific attention as the potential inhibitors of protein-protein interactions which are generally less druggable for small molecules (Dougherty et al., 2017).

Ivermectin (IVM), an artificial 16-member macrocyclic lactone, is derived from a mixture of avermectins B1a and B1b produced by the actinomycete *Streptomyces avermitili*s through fermentation (Burg et al., 1979; Ōmura, 2002). IVM comprises the principal dihydroavermectin B1a (IVM B1a) and the dihydroavermectin B1b at an imprecise ratio of 4:1 (Chabala et al., 1980). IVM has been widely used as a nematocidal, acaricidal, and insecticidal compound in agriculture and veterinary science since 1981 (Crump, 2017). In 1987, it was first approved for use in humans to treat onchocerciasis (river blindness) caused by the blackfly-transmitted parasite *Onchocerca volvulus* (Crump, 2017; Ottesen and Campbell, 1994). It is commercially available also for other human diseases such as lymphatic filariasis, strongyloidiasis, scabies, and head lice (Crump, 2017). In addition to its approved uses, IVM is expected to be effective against viruses, including severe acute respiratory syndrome coronavirus 2 (SARS-CoV-2) (Caly et al., 2020; Heidary and Gharebaghi, 2020), and various cancers (Tang et al., 2021).

IVM binds to glutamate-gated chloride channels and potentiates them in the nerve and muscle cells of parasites, causing neuronal hyperpolarization, inducing muscle paralysis, and subsequently killing the parasites (Arena et al., 1992; Cully et al., 1994). In contrast, to our knowledge, the direct targeting of other biological activities in viruses and mammals by IVM has not been reported. Although importins (IPOs) are viable candidate targets for the antiviral activity of IVM (Wagstaff et al., 2012), the physical interaction between IVM and IPOs remains unverified. On the other hand, the anticancer activities of IVM involve inhibitions of the Wnt/β-catenin and AKT/mechanistic target of rapamycin (mTOR) pathways and programmed cell death (Tang et al., 2021). However, the mammalian targets of IVM are yet to be identified.

The Wnt/β-catenin pathway plays a crucial role in the self-renewal of stem cells and maintenance of homeostasis (Clevers and Nusse, 2012). However, aberrant Wnt/β-catenin signaling causes a wide range of human diseases (Clevers and Nusse, 2012). Moreover, abnormal activation of the Wnt/β-catenin and AKT/mTOR pathways is frequently observed in various cancers, including colorectal cancer; therefore, the components of these pathways constitute attractive therapeutic targets (Cheng et al., 2019; Yang et al., 2019). The accumulation of β-catenin in the cytoplasm and subsequent regulation of gene expression with a transcription factor, TCF4, in the nucleus are the central events for the Wnt/β-catenin signaling pathway. On the other hand, the AKT/mTOR pathway, following phosphatidylinositol 3-kinase (PI3K) activation, regulates several cellular behaviors such as growth, proliferation, metabolism, protein and lipid synthesis, and autophagy (Laplante and Sabatini, 2012). Deviance from the normal mTOR regulation has often been reported in cancer pathology (Laplante and Sabatini, 2012). Furthermore, a close association has been identified between the Wnt/β-catenin and PI3K/AKT/mTOR pathways. Indeed, these signaling pathways regulate each other and share common signaling molecules, including glycogen synthase kinase 3 (GSK3) (Prossomariti et al., 2020).

This study rediscovered IVM as an inhibitor of Wnt/β-catenin signaling (Melotti et al., 2014), inducing unconventional β-catenin degradation even in the presence of proteasomal inhibitors. We further identified telomere length regulation protein 2 homolog (TELO2)—a regulator of phosphatidylinositol 3-kinase-related kinases (PIKKs), including mTOR complexes 1/2 (mTORC1/2)—as a mammalian target of IVM. The binding of IVM B1a to TELO2 mediated the inhibition of Wnt/β-catenin signaling. IVM reduced TELO2 and PIKK protein levels and inhibited mTOR downstream signaling, leading to the reduction of the β-catenin level. Our data indicate that TELO2 serves as the interconnection between the Wnt/β-catenin and AKT/mTOR pathways. Therefore, it can be a druggable target molecule for multiple human diseases.

## Results

### A screening for chemical suppressors of the eyeless phenotype identified IVM as an inhibitor of the Wnt/β-catenin pathway

**Figure 1:**
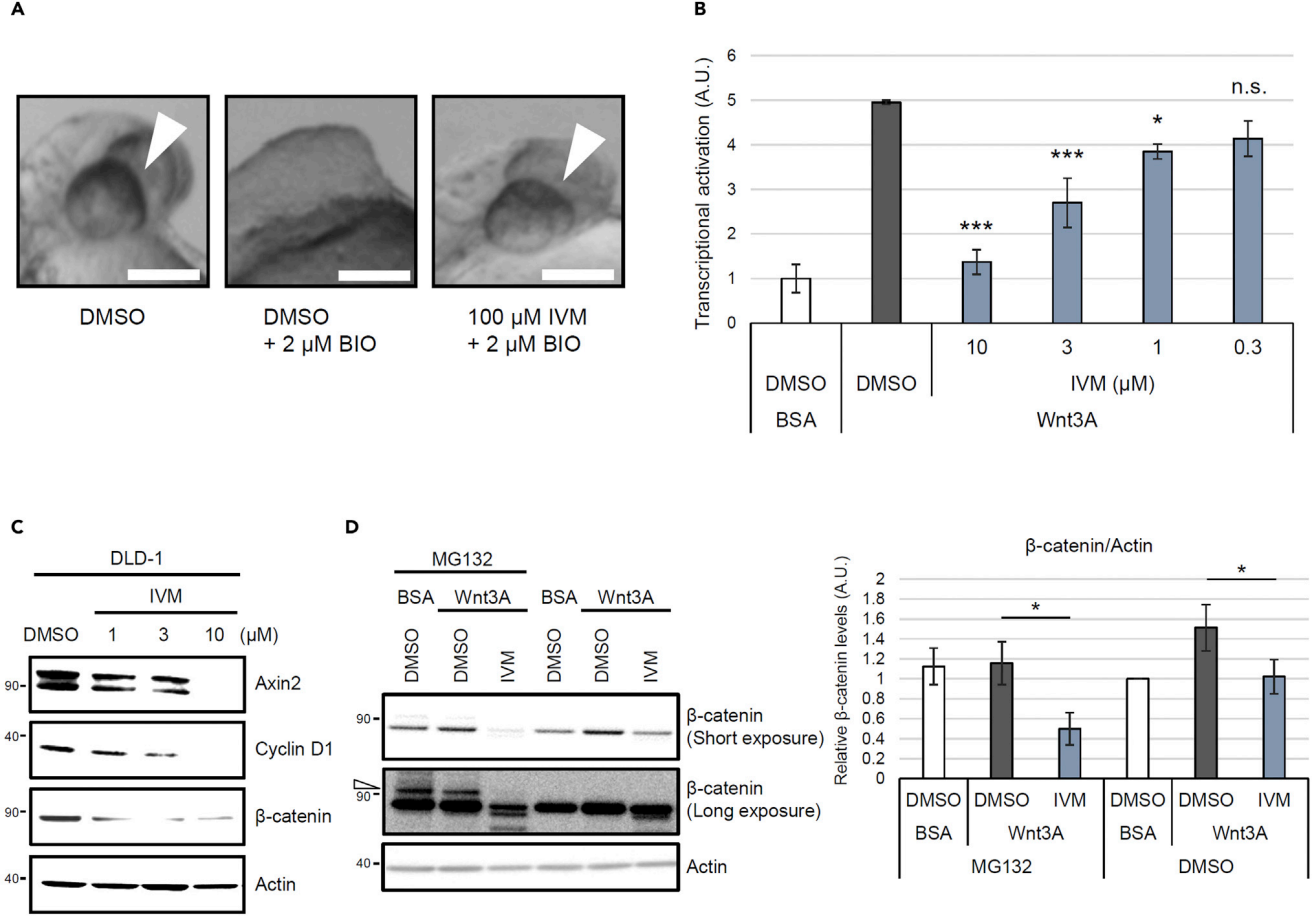
Ivermectin (IVM) suppresses the Wnt/β-catenin pathway in zebrafish embryos and mammalian cells (A) IVM was identified as a chemical suppressor of the eyeless phenotype. Zebrafish embryos were pretreated with 100 μM IVM at 50% epiboly. They were then treated with 6-bromo-indirubin-3′-oxime (BIO), which is a GSK3 inhibitor that leads to the accumulation of β-catenin, at the shield stage and were incubated for 24 h. Images were obtained at 30 h postfertilization. Scale bar = 200 μm. See also Figure S1. (B) IVM reduced the β-catenin/TCF-dependent transcriptional activity. Human embryonic kidney 293 (HEK293) cells were transiently cotransfected with Super 8x TOPFlash—a firefly luciferase reporter plasmid—to monitor the β-catenin/TCF*-*dependent transcriptional activity and with pRL-SV40—a renilla luciferase reporter plasmid—to normalize the transfection efficiency. The cells were pretreated with IVM at the indicated concentrations for 1 h and then treated with 50 ng/mL of Wnt3A for 18 h in 1% fetal bovine serum (FBS)–supplemented Dulbecco’s Modified Eagle Medium (DMEM). Transcriptional activation in the cells was assayed by measuring the firefly and renilla luciferase activities. Normalized relative luciferase activities were calculated by dividing firefly luciferase activities by those of renilla. Transcriptional activation levels are indicated as values relative to the control (dimethyl sulfoxide [DMSO]-treated and bovine serum albumin [BSA]–treated cells). Data are presented as the means ± standard errors of the means (n = 4 biological replicates). ∗p < 0.05, ∗∗∗p < 0.001, n.s.: not significant, one-way ANOVA with Tukey’s test. (C) IVM downregulated the target proteins involved in Wnt/β-catenin signaling. Human colorectal cancer DLD-1 cells were treated with IVM at the indicated concentrations for 18 h in 1% FBS-supplemented RPMI 1640 medium. Subsequently, the cytoplasmic fractions of the cells were probed for Axin2, cyclin D1, β-catenin, and actin. (D) IVM reduced the cytoplasmic β-catenin levels in the presence of a proteasomal inhibitor, MG132. HEK293 cells were first treated with 25 μM MG132 for 15 min, followed by treatment with 10 μM IVM for 1 h and 50 ng/mL of Wnt3A for 2 h in 1% FBS/DMEM. The cytoplasmic proteins were probed with anti-β-catenin and anti-actin antibodies (the left panel). The band intensities were quantified, normalized to the actin levels, and reported as values relative to the control (DMSO-treated and BSA-treated cells in the absence of MG132; right panel). The open triangle indicates the bands of ubiquitinated β-catenin. Data are presented as the means ± standard deviations (SDs; n = 3 biological replicates). ∗p < 0.05, one-way ANOVA with Tukey’s test. See also .Figure S2

Through our screening for chemical suppressors of the eyeless phenotype—the hallmark of uncontrolled activation of Wnt/β-catenin signaling—in zebrafish embryos, we rediscovered IVM as an inhibitor of the Wnt/β-catenin pathway. Aberrant activation of Wnt/β-catenin signaling by 6-bromo-indirubin-3′-oxime (BIO), a GSK3 inhibitor that leads to the accumulation of β-catenin, resulted in the eyeless phenotype (Nishiya et al., 2014). The eye development was restored by the 100 μM IVM (Figure 1A). IVM led to eye development restoration at a concentration of 12.5–100 μM without inducing an additional phenotype in the tail (Figure S1), indicating the existence of substantial differences between the effective concentration for suppressing the aberrant Wnt/β-catenin signaling activation and the toxic concentration. The activity of IVM was confirmed in human cell lines in addition to zebrafish (Figures 1B–1D). Although the inhibitory effect of IVM on the Wnt/β-catenin pathway has been reported in a previous study (Melotti et al., 2014), the direct target and precise action mechanism of IVM remain elusive. IVM has demonstrated proteasome-independent β-catenin reduction in the presence of proteasome inhibitors MG132 (Figure 1D) or lactacystin (Figure S2A) (Ōmura and Crump, 2019). Although MG132 or lactacystin triggered accumulation of the higher-molecular-weight ubiquitinated forms of β-catenin (Aberle et al., 1997), IVM reduced the level of the ubiquitinated form in addition to that of the nonubiquitinated form of the protein (Figures 1D and S2A). In contrast, the degradation of β-catenin induced by XAV939, which is a well-characterized inhibitor of Wnt/β-catenin signaling, was completely blocked by MG132 (Figure S2B). These data suggest that IVM inhibits Wnt/β-catenin signaling through an unconventional mechanism.

### TELO2 regulates the Wnt/β-catenin signaling pathway

**Figure 2:**
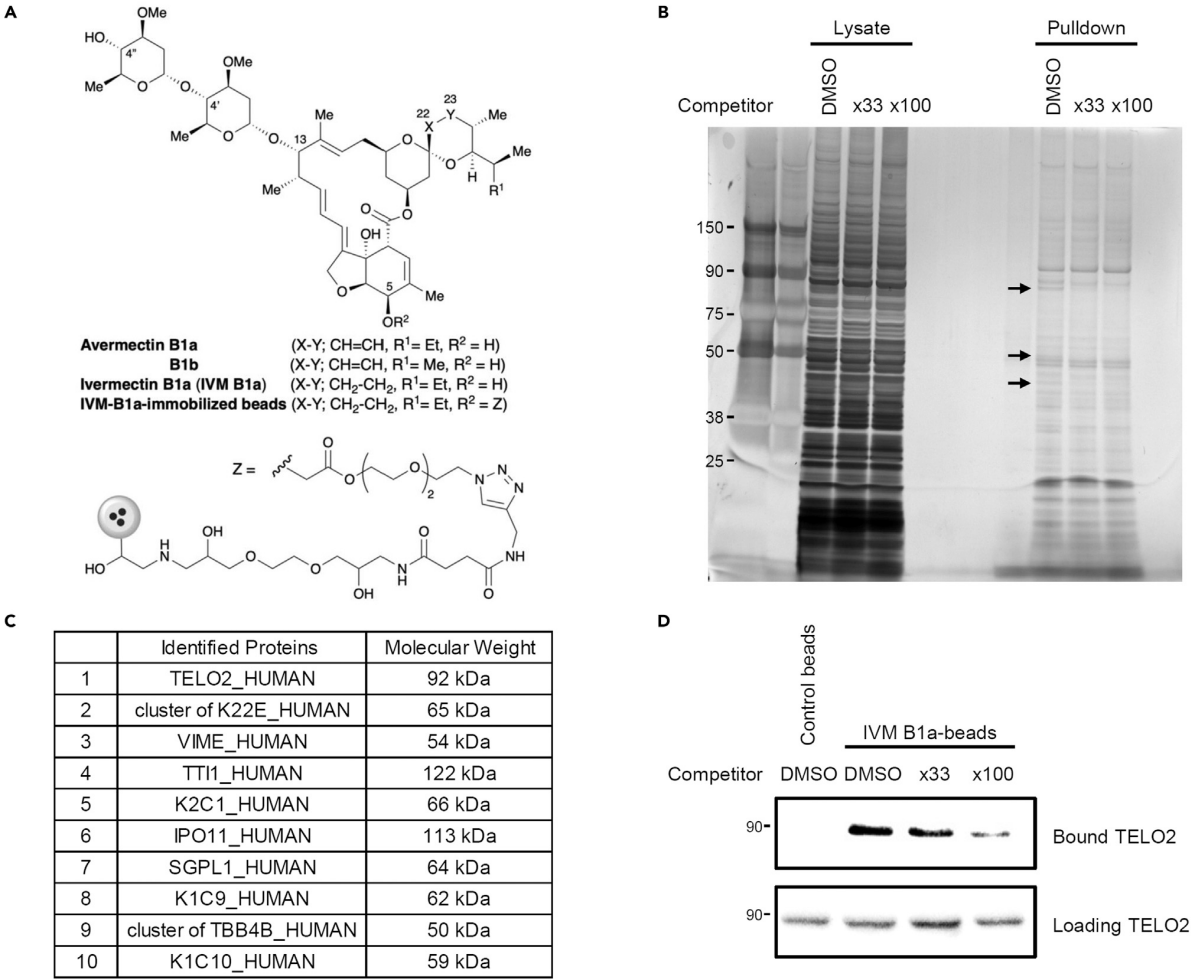
Identification of telomere length regulation protein 2 homolog (TELO2) as a mammalian target of IVM (A) Chemical structure of avermectins and immobilized dihydroavermectin B1a (IVM B1a) for target identification. (B) IVM B1a-binding proteins were identified through a pull-down assay with chemically immobilized IVM B1a. HEK293 cell lysates were incubated with the affinity beads in the absence or presence of soluble IVM as a competitor at 33- or 100-folds. The bound fractions were eluted, resolved by SDS-PAGE, and visualized with silver staining. The arrows indicate the bands that were competed out by the competitor. (C) Mascot analysis of the data was summarized. TELO2 was identified as an IVM B1a-binding protein. Cluster of K22E, keratins, type II cytoskeletal 2 epidermal, 5, 6A, 6B, and 75; VIME, vimentin; TTI1, TELO2-interacting protein 1 homolog; K2C1, keratin, type II cytoskeletal 1; IPO11, importin-11; SGPL1, sphingosine-1-phosphate lyase 1; K1C9, keratin, type I cytoskeletal 9; cluster of TBB4B, cluster of tubulin beta, beta-2A, beta-4B, and beta-6 chains; K1C10, keratin, type I cytoskeletal 10. See also Data S1. (D) Binding of TELO2 to the immobilized IVM B1a was confirmed through western blotting with an anti-TELO2 antibody. Free IVM was added as the competitor.

**Figure 3:**
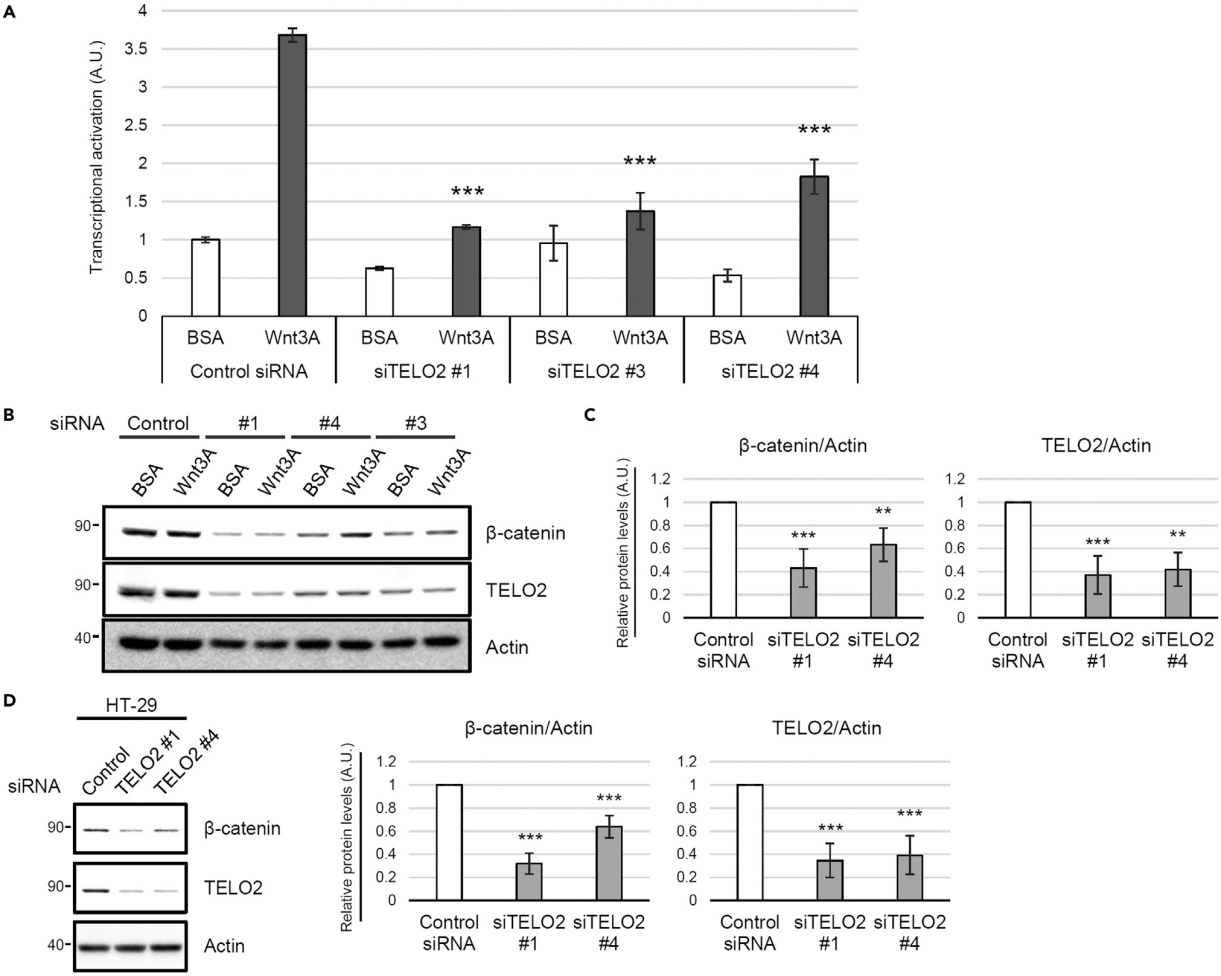
TELO2 is essential for the maintenance of β-catenin levels (A) Knockdown of *TELO2* with siRNAs reduced β-catenin/TCF-dependent transcriptional activation in HEK293 cells. The cells transfected with control or *TELO2* siRNAs, and then transiently cotransfected with Super 8x TOPFlash (a firefly luciferase reporter plasmid) to assess the β-catenin/TCF*-*dependent transcriptional activity and with pRL-SV40 (a renilla luciferase reporter plasmid) to normalize the transfection efficiency. The cells were treated with 50 ng/mL Wnt3A for 18 h. Firefly and renilla luciferase activities were measured. Normalized relative luciferase activities were calculated by dividing firefly luciferase activities by those of renilla and indicated the level of transcriptional activation. Data are presented as the means ± SDs (n = 3 biological replicates). ∗∗∗p < 0.001, one-way ANOVA with Tukey’s test. (B–D) Knockdown of *TELO2* with siRNAs reduced the β-catenin levels in HEK293 and HT-29 cells. HEK293 (B and C) or HT-29 (D) cells were transfected with siRNAs for *TELO2* for 72 h. The cell lysates were analyzed via western blotting with the indicated antibodies (B and D). (C and D) Band intensities were quantified, normalized to the actin levels, and reported as values relative to those obtained from the cells transfected with a control siRNA. Data are presented as the means ± SDs (C, n = 3 biological replicates; D, n = 4 biological replicates). ∗∗p < 0.01, ∗∗∗p < 0.001, one-way ANOVA with Tukey’s test.

We screened for the direct targets of IVM for the inhibition of the Wnt/β-catenin pathway by preparing IVM B1a-immobilized beads (Figure 2A). The bound fraction from human embryonic kidney 293 (HEK293) cell lysate included proteins that could be competed out by adding free IVM as a competitor (Figure 2B). Mass spectrometry followed by Mascot analysis revealed the identities of these specifically bound proteins. TELO2 showed 100% protein identification probability and the highest percent coverage (63%) except for keratin and vimentin. In addition to TELO2 itself, its binding partners TTI1 and TTI2 were detected among the bound proteins (Figure 2C and Data S1). Western blotting with an anti-TELO2 antibody in the presence or absence of free IVM confirmed specific binding of TELO2 to IVM (Figure 2D). Furthermore, *TELO2* knockdown with siRNAs resulted in the inhibition of Wnt-induced β-catenin/TCF-dependent transcriptional activation (Figure 3A) and reduction of the β-catenin level in HEK293 (Figures 3B and 3C) and human colorectal adenocarcinoma (HT-29; Figure 3D) cells. These results indicate that TELO2 is involved in the Wnt/β-catenin signaling pathway.

### IVM B1a interacts with TELO2 via the C-terminal α-helix *in vitro* and in living cells

**Figure 4:**
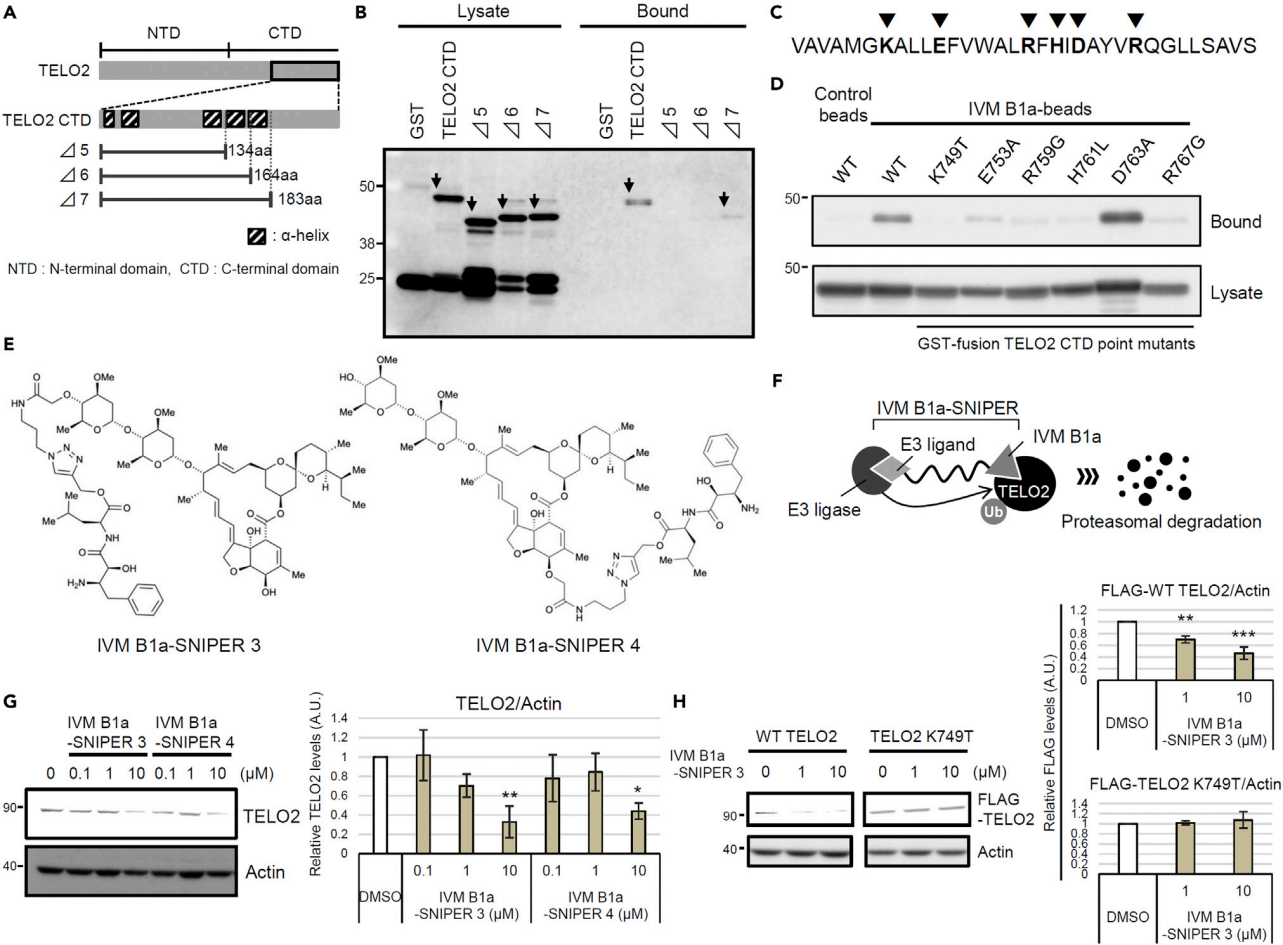
TELO2 requires the C-terminal α-helix integrity for its binding to IVM B1a (A) TELO2 deletion mutants lacking α-helices in the C-terminal region. (B) Glutathione S-transferase (GST)-fusion TELO2 deletion mutants Δ5 and Δ6 lost the binding affinity for IVM B1a. Lysates from *E. coli* expressing GST-fusion TELO2 mutants were subjected to the binding assay. The lysates and the bound fractions were analyzed through western blotting with an anti-GST antibody. Arrows indicate the bands corresponding to TELO2 mutants. (C) Sites of point mutations in the C-terminal α-helix are indicated as triangles. (D) Site-directed mutagenesis in the C-terminal helix of TELO2 inhibited the binding of TELO2 to IVM B1a. Binding affinities of the point mutants were analyzed by probing the lysates and bound fractions with an anti-GST antibody. (E) Chemical structures of IVM B1a-SNIPERs (specific and nongenetic IAP-dependent protein erasers) 3 and 4. (F) IVM B1a-SNIPER promotes the proteasomal degradation of TELO2. (G) IVM B1a-SNIPERs 3 and 4 reduced endogenous TELO2. HEK293 cells were treated with IVM B1a-SNIPERs 3 or 4 for 18 h in 1% FBS/DMEM. Then, the cell lysates were probed with anti-TELO2 and anti-actin antibodies (the left panel). The band intensities were quantified, normalized to the actin levels, and indicated as values relative to the control (DMSO) (the right panel). Data are presented as the means ± SDs (n = 3 biological replicates). ∗p < 0.05, ∗∗p < 0.01, one-way ANOVA with Tukey’s test. See also Figure S3. (H) Interaction of IVM B1a with TELO2 depended on K749 in cells. HEK293 cells were transfected with vectors encoding FLAG-tagged WT or K749T TELO2 and treated with 1 or 10 μM IVM B1a-SNIPER 3 for 5 h in 1% FBS/DMEM. Cell lysates were probed with anti-FLAG and anti-actin antibodies (the left panel). The band intensities were quantified, normalized to the actin levels, and indicated as values relative to the control (DMSO; the right panels). Data are presented as the means ± SDs (n = 3 biological replicates). ∗∗p < 0.01, ∗∗∗p < 0.001, one-way ANOVA with Tukey’s test. See also Figure S4.

We studied the binding mode between TELO2 and IVM by generating a deletion mutant expression system in *Escherichia coli* (Figure 4A). The binding ability of TELO2 to the IVM B1a beads was greatly reduced in the TELO2 Δ6 mutant, which lacked the last C-terminal helix structure of the multiple α-helices in TELO2 (Figures 4A and 4B). Introduction of single amino acid substitutions in the C-terminal helix, such as in the cases of TELO2 K749T, E753A, R759G, H761L, and R767G, resulted in the TELO2 mutants losing their *in vitro* IVM B1a-binding abilities (Figures 4C and 4D).

We examined the physical interaction between TELO2 and IVM in living cells using a protein knockdown technique *specific and nongenetic inhibitor of apoptosis protein-dependent protein eraser* (SNIPER) (Ohoka et al., 2016). IVM B1a-SNIPERs are chimeric compounds comprising IVM B1a connected to ubenimex—a ligand for the cellular inhibitor of apoptosis protein 1 (cIAP1) ubiquitin ligase—via a linker (Figures 4E and S3A). We anticipated that IVM B1a-SNIPERs induced the proteasomal degradation of IVM B1a-binding proteins that were drawn into the closeness, proximity of cIAP1 (Figure 4F). IVM B1a-SNIPERs 3 and 4 reduced the TELO2 level in a concentration-dependent manner (Figure 4G), whereas IVM B1a-SNIPERs 1 and 2 did not (Figure S3B). These data suggest that TELO2-binding to IVM is independent of the hydroxy group of the disaccharide and the C5-hydroxyl group of the benzofuran ring and that the optimal linker length is crucial for the degradation induction. IVM B1a-SNIPER 3 reduced the level of FLAG-tagged wild-type (WT) TELO2 (FLAG-WT TELO2) but not that of FLAG-TELO2 K749T, which lacked IVM-binding ability (Figure 4H). MG132 prevented the IVM B1a-SNIPER-dependent degradation of TELO2 (Figure S4), indicating that IVM B1a-SNIPER 3 depends on proteasomal protein degradation. These data suggest that IVM B1a physically interacts with TELO2 in living cells.

### IVM reduces the β-catenin protein level by binding to TELO2

**Figure 5:**
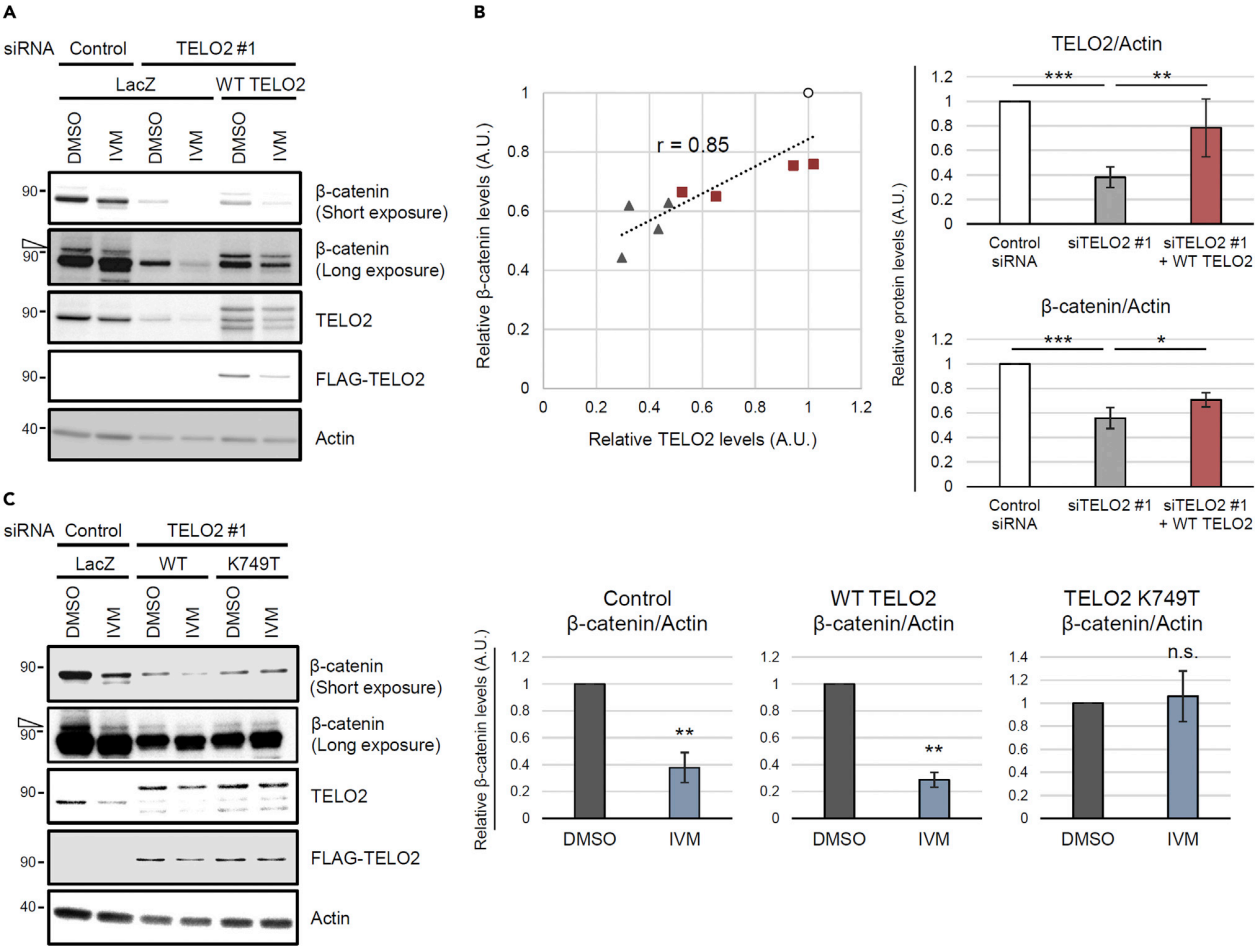
IVM suppresses Wnt/β-catenin signaling via binding to TELO2 (A and B) Reconstitution of the TELO2 restored β-catenin levels in TELO2-knockdown cells. HEK293 cells were transfected with *TELO2* siRNA for 4 days and transfected with an siRNA-resistant FLAG-tagged WT TELO2 expression vector. The cells were treated with 10 μM IVM for 1 h and then with 50 ng/mL Wnt3A for 2 h in the presence of 25 μM MG132. (A) Cell lysates were analyzed through western blotting with anti-β-catenin, anti-TELO2, anti-FLAG, and anti-actin antibodies. The open triangle indicates the bands corresponding to ubiquitinated β-catenin. (B) Band intensities of TELO2 and β-catenin were quantified in control siRNA-transfected (circles), TELO2 #1 siRNA-transfected (triangles), or TELO2-reconstituted (squares) cells in the absence of IVM, normalized to the actin levels, and indicated as values relative to the control siRNA-transfected cells. The X- and Y axes of the left panel indicate relative TELO2 and β-catenin levels, respectively (n = 4 biological replicates). Correlation coefficient (*r*) = 0.85. Data of the right panels represent the means ± SDs (n = 4 biological replicates). ∗p < 0.05, ∗∗p < 0.01, ∗∗∗p < 0.001, one-way ANOVA with Tukey’s test. (C) TELO2 K749T reconstitution conferred IVM resistance. *TELO2*-knockdown HEK293 cells were transfected with vectors expressing siRNA-resistant FLAG-tagged WT TELO2 or TELO2 K749T. The cells were treated with 25 μM MG132 for 15 min, 10 μM IVM for 1 h, and 50 ng/mL Wnt3A for 2 h in 1% FBS/DMEM. Protein levels were analyzed through western blotting with specific antibodies (the left panel). The open triangle indicates bands corresponding to ubiquitinated β-catenin. The band intensities were quantified, normalized to actin levels, and indicated as values relative to the control (DMSO) (the right panels). Data are presented as the means ± SDs (n = 3 biological replicates). ∗∗p < 0.01, n.s.: not significant, Welch's *t*-test. See also .Figure S5

As *TELO2* knockdown reduced the β-catenin level (Figures 3B–3D), we investigated whether reconstitution with exogenous TELO2 restores the level of β-catenin in *TELO2*-knockdown cells. The β-catenin level could be regulated through the conventional proteasomal degradation in addition to the abovementioned unconventional proteasome-independent mechanism (Figures 1D and S2A). To avoid the confounding effects of the proteasomal activity induced by the cellular stress caused by multistep experimental procedures, the reconstitution experiments were performed in the presence of MG132. Transfecting the knockdown cells with a plasmid that encodes siRNA-resistant WT TELO2 restored the β-catenin level (Figures 5A and 5B). The endogenous and exogenous TELO2 protein levels showed strong positive correlation with that of β-catenin (r = 0.85, Figure 5B). To assess whether the binding of IVM to TELO2 mediates its pharmacological inhibition of the Wnt/β-catenin pathway, sensitivities to IVM were compared between the WT TELO2 and K749T mutant. IVM reduced nonubiquitinated and ubiquitinated β-catenin levels in cells reconstituted with FLAG-WT TELO2 (Figures 5A and 5C), whereas reconstitution with FLAG-TELO2 K749T was resistant to IVM-induced β-catenin degradation (Figure 5C). The same tendency was observed in the β-catenin/TCF reporter assay. Although *TELO2* knockdown reduced β-catenin/TCF-dependent transcriptional activation, reconstitutions with FLAG-WT TELO2 or FLAG-TELO2 K749T restored it (Figure S5). IVM significantly reduced transcriptional activation in the control and WT TELO2-reconstituted cells. However, the cells reconstituted with TELO2 K749T were less sensitive to IVM compared with WT TELO2-reconstituted cells (Figure S5), further confirming that IVM inhibited Wnt/β-catenin signaling via its binding to TELO2.

### IVM reduces TELO2 and negatively regulates PIKKs

**Figure 6:**
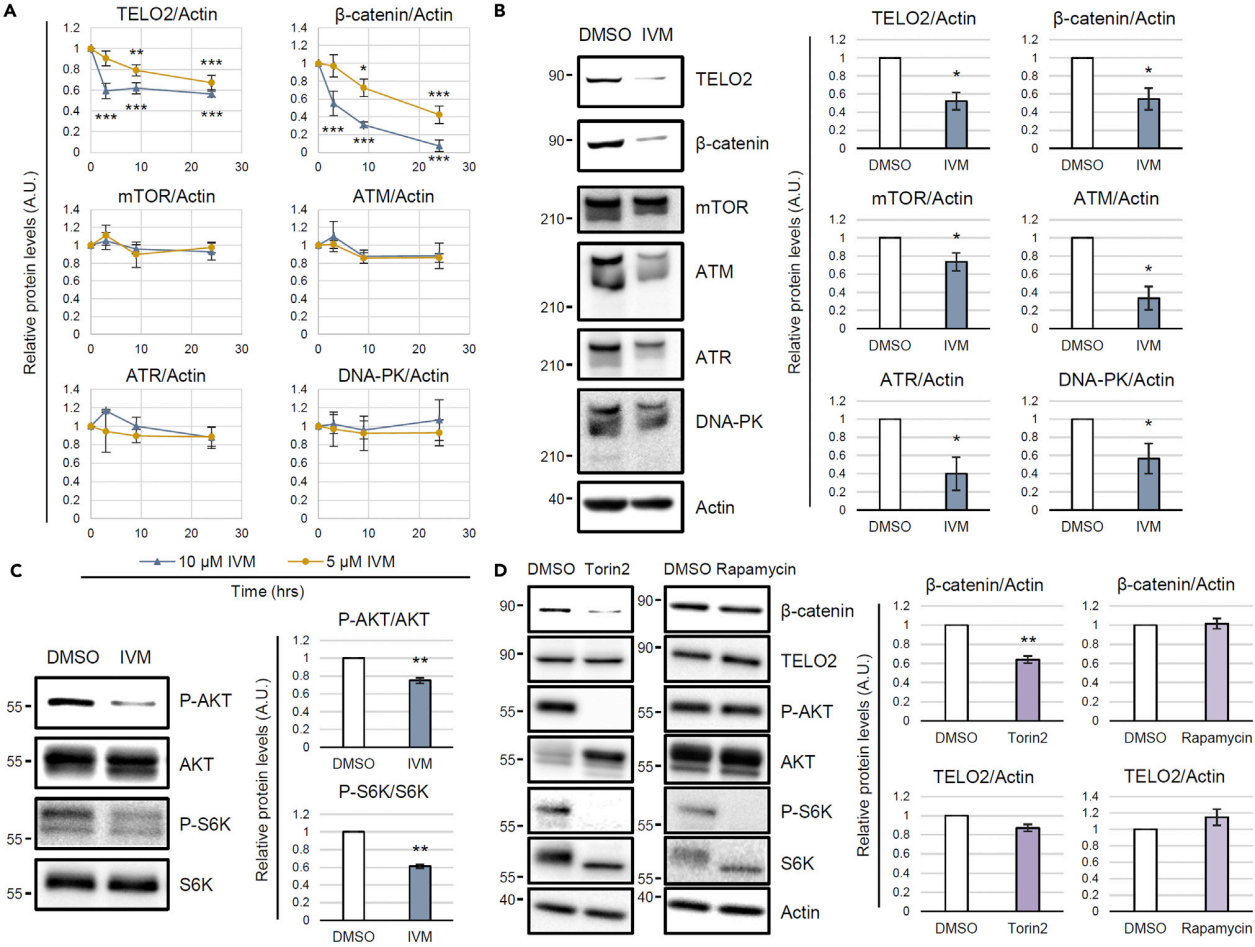
IVM reduces TELO2, phosphatidylinositol 3-kinase-related kinase (PIKK) levels and mTOR substrate phosphorylation levels (A) Short-term IVM treatment reduced TELO2 and β-catenin, but not PIKK levels. HEK293 cells were treated with 5 or 10 μM of IVM for 3, 9, or 24 h in 1% FBS/DMEM. Subsequently, the cell lysates were probed for TELO2, cytoplasmic β-catenin, mTOR, ataxia telangiectasia mutated (ATM), ATM-related and Rad3-related (ATR), DNA-dependent protein kinase (DNA-PK), and actin through western blotting with specific antibodies. The band intensities were quantified, normalized to the actin levels, and indicated as values relative to the control (0 h). Data are presented as the means ± SDs (n = 3 biological replicates). ∗p < 0.05, ∗∗p < 0.01, ∗∗∗p < 0.001, one-way ANOVA with Tukey’s test. (B) Long-term IVM treatment reduced PIKKs. HEK293 cells were treated with 5 μM IVM for 72 h in 1% FBS/DMEM. The cell lysates were probed through western blotting with the indicated antibodies (the left panels). The band intensities were quantified, normalized to the actin levels, and indicated as values relative to the control (DMSO) (the right panels). Data are presented as the means ± SDs (n = 3 biological replicates). ∗p < 0.05, Welch’s *t*-test. (C) Short-term IVM treatment reduced AKT and S6 kinase phosphorylation levels. HEK293 cells were treated with 10 μM IVM for 3 h in 1% FBS/DMEM. The cell lysates were probed through western blotting with the indicated antibodies (left panels). The band intensities were quantified, normalized by the total protein levels, and indicated as values relative to the control (DMSO) (the right panels). Data are presented as the means ± SDs (n = 3 biological replicates). ∗∗p < 0.01, Welch’s *t-*test. See also Figures S6 and S7. (D) Torin2 reduced the cytoplasmic level of β-catenin. HEK293 cells were treated with 0.1 μM Torin2 or rapamycin for 3 h in 1% FBS/DMEM. The cytoplasmic proteins were probed through western blotting with the indicated antibodies (the left panels). The band intensities were quantified, normalized to the actin levels, and indicated as values relative to the control (DMSO; the right panels). Data are presented as the means ± SDs (n = 3 biological replicates). ∗∗p < 0.01, Welch’s *t-*test. See also Figure S8.

The IVM treatment reduced the level of TELO2 in addition to β-catenin (Figures 5A and 5C). Notably, this reduction in the TELO2 level caused by IVM was observed in the presence of MG132 (Figures 5A and 5C), whereas IVM-SNIPER 3 was sensitive to MG132 (Figure S4), indicating that IVM and IVM-SNIPERs reduced the TELO2 level through fundamentally different mechanisms. The native IVM-mediated intrinsic negative regulation of TELO2 might account for the mechanism of action of IVM. To analyze TELO2 kinetics, the protein levels were monitored during the IVM treatment. TELO2 reduction started within 3 h and reached approximately 50% by 72 h (Figures 6A and 6B). TELO2 is an essential cofactor of PIKKs, such as mTOR, ataxia telangiectasia mutated (ATM), ATM and Rad3-related (ATR), and DNA-dependent protein kinase (DNA-PK). IVM-induced TELO2 reduction might reduce the PIKK levels because TELO2 knockout or knockdown destabilizes PIKKs (Kaizuka et al., 2010; Takai et al., 2007, 2010). We also monitored the PIKK kinetics in the presence of 5 or 10 μM IVM. The long-term (72 h) treatment significantly reduced PIKK levels (Figure 6B). However, the short-term treatment did not affect these levels even if the TELO2 and β-catenin levels were reduced (Figure 6A). Therefore, the effect of IVM on β-catenin reduction should be mediated by a mechanism different from the absence of PIKKs.

Next, we evaluated the effect of short-term IVM treatment on the formation of a complex among TELO2, TTI1, mTOR, and ATM. FLAG-tagged WT TELO2 or the K749T mutant were immunoprecipitated by an anti-FLAG antibody, and the coprecipitated proteins were analyzed upon one-hour IVM treatment, during which the levels of the FLAG-tagged TELO2 proteins did not show any reduction. The physical interaction of TELO2 with its binding proteins remained intact even in the presence of 10 μM IVM both in the WT or IVM-resistant mutant (Figure S6). However, short-term IVM treatment significantly reduced the phosphorylation levels of AKT and S6 kinase, downstream molecules of PIKKs, including mTOR in HEK293 (Figure 6C) and HT-29 (Figure S7) cells. Moreover, short-term treatment of the mTOR kinase inhibitor Torin2 reduced the β-catenin level without affecting that of TELO2, whereas an mTORC1-specific inhibitor, rapamycin, did not affect the β-catenin level (Figure 6D). Rapamycin did not preclude IVM-induced β-catenin reduction (Figure S8), although mTORC1 inhibition prevents a feedback loop and paradoxically activates AKT (Peterson et al., 2009; Wang et al., 2007).

## Discussion

In this study, we showed that IVM suppresses Wnt/β-catenin signaling by binding to TELO2 that is essential for the maintenance and functions of PIKKs.

TELO2 physically interacts with IVM. TELO2 was identified as IVM B1a-binding protein along with its binding partners TTI1 and TTI2 (Takai et al., 2010) through affinity purification and mass spectrometry (Figure 2C and Data S1). The C-terminal α-helix of TELO2 played an indispensable role in binding of IVM B1a *in vitro* (Figures 4B and 4D). Targeted degradation of TELO2 by IVM B1a-SNIPER 3 and 4 confirmed the interaction in living cells (Figures 4G and 4H). In *Caenorhabditis elegans*, IVM binds to the transmembrane domain of the glutamate-gated chloride channel (GluCl) comprising five homologous subunits, each harboring four α-helical transmembrane spans. IVM reaches deeply into the interface between two subunits and forms contact with packed α-helices (Hibbs and Gouaux, 2011). Likewise, TELO2 has helical repeats in which the neighboring helices are packed into a superhelical structure, α-solenoid (Takai et al., 2010). The C-terminal solenoid of TELO2 exhibits substantial structural similarities to proteins possessing helical structures, such as IPOs, A subunit of protein phosphatase 2A (PP2A), cullin homolog 1, and microtubule-binding proteins (Takai et al., 2010). IPOs were repeatedly found in the list of the putative IVM-binding proteins (Figure 2C and Data S1) in this study. However, the A subunit of PP2A did not bind to the IVM B1a affinity resin under our experimental conditions and was absent in the list (Data S1). Furthermore, site-directed mutagenesis revealed essential amino acid residues (Figures 4C and 4D), suggesting that contacts with specific amino acid residues rather than the overall structure or the hydrophobic features of helical structures were crucial for a protein’s affinity to bind to IVM B1a.

The introduction of ubenimex in the IVM B1a, such as SNIPERs 3 and 4, did not influence the binding between the IVM B1a moiety and TELO2. Thus, the binding mode of IVM B1a to TELO2 is likely different from that to GluCls, which depends on the C5-hydroxyl group of the benzofuran ring (Hibbs and Gouaux, 2011); in addition, the spiroacetal moiety may be a target interface. IVM inhibits IPO-mediated nuclear localization of viral proteins (Wagstaff et al., 2012); it has broad antiviral effects on several viruses, including SARS-CoV-2 (Tay et al., 2013; Wagstaff et al., 2012; Caly et al., 2020; Heidary and Gharebaghi, 2020; Yang et al., 2020). Therefore, the derivatization of IVM may produce anticancer and antiviral drugs with different target-specificity for TELO2 and IPOs. Precise structural analysis of the TELO2–IVM complex may give a direction of derivatization during the development of safe and potent second-generation IVM molecules with increased specificity and binding affinity.

Our data suggest that TELO2 reduction mediates the IVM-induced suppression of Wnt/β-catenin signaling. *TELO2* knockdown reduced the β-catenin levels and β-catenin/TCF-dependent transcriptional activation (Figure 3). The reconstitution system with WT TELO2 restored the level of β-catenin (Figure 5) as well as the transcriptional activation (Figure S5). The cells reconstituted with TELO2 K749T, a mutant without IVM B1a-binding ability, were resistant to IVM, whereas those reconstituted with WT TELO2 were sensitive (Figures 5C and S5). IPO11, an IVM-binding protein (Figure 2C and Data S1), is also involved in the nuclear import of β-catenin (Mis et al., 2020). The existence of other IVM targets might explain the partial resistance of TELO2 K749T to IVM in the β-catenin/TCF-dependent transcriptional activation (Figure S5). Moreover, IVM reduced the level of the WT TELO2 but not that of the K749T mutant in the presence of MG132 (Figure 5C), suggesting that the binding of IVM to TELO2 induces TELO2 degradation in a proteasome-independent manner. TELO2 is an important cofactor for PIKKs such as mTOR, ATM, ATR, and DNA-PK (Kaizuka et al., 2010; Takai et al., 2007, 2010). In this study, long-term IVM treatment reduced the protein levels of PIKKs (Figure 6B). In particular, mTOR has been proposed as an intersection between the Wnt/β-catenin and PI3K/AKT signaling pathways. Indeed, ATP-competitive mTOR kinase inhibitors, PP242 (Petherick et al., 2013) and Torin2 (this study), suppressed β-catenin/TCF-dependent transcriptional activation and β-catenin protein levels, respectively. In this study, IVM reduced the phosphorylation levels of AKT and S6 kinase (Figures 6C and S7), which was consistent with the results of IVM treatment or *TELO2* knockdown reported in previous studies (Dou et al., 2016; Kaizuka et al., 2010). The suppression of mTOR signaling results in autophagy and apoptosis (Laplante and Sabatini, 2012). Autolysosome (Petherick et al., 2013) and caspase-3 (Steinhusen et al., 2000) are involved in nonproteasomal β-catenin degradation. These mechanisms are candidates of the IVM-induced β-catenin degradation. On the other hand, PP2A reportedly mediates the anti-Wnt signaling activity of IVM (Melotti et al., 2014). The direct interaction between IVM and the A subunit of PP2A was not detected in this study. However, our data do not necessarily exclude the PP2A-mediated mechanism. The involvement of a common signaling factor among TELO2 and PP2A, such as mTOR, may account for the molecular link between them. Thus, the functional modulation of TELO2, a component of mTORCs, mediates the suppression of Wnt/β-catenin signaling through IVM.

In this study, proteasome inhibition tends to enhance β-catenin degradation by IVM (Figures 1D and S2A). Proteasome inhibitors initiate the unfolded protein response, eventually causing ER stress-induced apoptosis (Obeng et al., 2006). Therefore, proteasome inhibitors might have enhanced the IVM-induced autophagic or apoptotic responses because of TELO2 disruption. Furthermore, IVM inhibits sarcoplasmic/ER Ca^2+^-ATPase (SERCA), leading to the generation of ER stress (Bilmen et al., 2002; Lytton et al., 1991). These multilayered stress responses may account for the enhanced β-catenin cleavage. Because ER stress inhibits the Wnt/β-catenin pathway (Costa et al., 2019), these findings suggest the combined use of IVM and a proteasome inhibitor to treat β-catenin-dependent cancers such as colorectal cancers. Although we focused on the modulation of TELO2 functions in the mTOR pathway, TELO2 is also essential for the maintenance of DNA-damage-responsive PIKKs (ATM, ATR, and DNA-PK). Therefore, combination therapies of IVM with conventional DNA-damaging anticancer drugs would be of interest in addition to proteasome inhibitors.

This study identified TELO2 as a mechanistic target of IVM in suppressing the Wnt/β-catenin signaling pathway. Our findings potentially pave the way for targeting of the nonenzymatic protein TELO2 that is crucial for stress-responsive PIKKs. TELO2 inhibition might simultaneously inactivate PIKKs and sensitize ER stress and DNA-damage, leading to immunogenic cell death. This immunity-related activity may help understand the mysterious long-lasting effects of IVM (Crump, 2017). These might facilitate the application of TELO2-targeting drugs in the development of cancer cell vaccines that can prevent cancer recurrence.

### Limitations of the study

This study demonstrated that IVM inhibits Wnt/β-catenin signaling through binding to TELO2, a component of the TELO2-TTI1-TTI2 complex indispensable for maintaining the levels and activities of PIKKs and that IVM reduces the levels of TELO2, β-catenin, and PIKKs. However, answers to the following questions remain to be explored in the future: (1) Which PIKKs account for IVM-induced β-catenin reduction? Torin2 inhibits all PIKKs, although mTOR is the most sensitive. (2) Would targeting other molecules with IVM suppress the Wnt/β-catenin signaling pathway? IVM-binding proteins include IPO11 and SERCA2, which may regulate different steps of the Wnt β-catenin signaling pathway in addition to the regulation of the β-catenin level by TELO2. Reconstitution with TELO2 K749T restored the protein level of β-catenin but partially reversed the β-catenin/TCF-dependent transcriptional activation in this study. The relative contributions of the target molecules of IVM that are involved in various steps of the Wnt/β-catenin pathway are yet to be determined. (3) What mediates β-catenin degradation in IVM-treated cells? Autolysosome and caspase-3 are involved in nonproteasomal β-catenin degradation (Petherick et al., 2013; Steinhusen et al., 2000). Our study revealed that IVM suppressed the downstream of mTOR signaling that prevents autophagy and apoptosis. Although autolysosomes and caspases are strong candidates, their contributions have not been investigated in this study. (4) The effect of IVM on DNA damage response has not been evaluated in this study.

## STAR★Methods

### Key resources table

**Table:** 

REAGENT or RESOURCE	SOURCE	IDENTIFIER
**Antibodies**		

Actin	Sigma-Aldrich	Cat# A4700; RRID: AB_476730
β-catenin	Sigma-Aldrich	Cat# C7207; RRID: AB_476865
GST-Tag	Proteintech	Cat# 66001-2-Ig; RRID: AB_2881488
TELO2	Proteintech	Cat# 15975-1-AP; RRID: AB_2203337
TTI1	Proteintech	Cat# 22381-1-AP; RRID: AB_2879094
Axin2	Cell Signaling Technology	Cat# 2151; RRID: AB_2062432
Cyclin D1	Cell Signaling Technology	Cat# 2926; RRID: AB_2070400
mTOR	Cell Signaling Technology	Cat# 2972; RRID: AB_330978
ATM	Cell Signaling Technology	Cat# 2873; RRID: AB_2062659
ATR	Cell Signaling Technology	Cat# 13934; RRID: AB_2798347
DNA-PK	Cell Signaling Technology	Cat# 12311; RRID: AB_2797881
Phospho-AKT (Ser473)	Cell Signaling Technology	Cat# 4060; RRID: AB_2315049
AKT	Cell Signaling Technology	Cat# 4691; RRID: AB_915783
Phospho-p70 S6 kinase (Thr389)	Cell Signaling Technology	Cat# 9206; RRID: AB_2285392
p70 S6 Kinase	Cell Signaling Technology	Cat# 9202; RRID: AB_331676
DDDDK-tag (FLAG-tag)	Medical & Biological Laboratories	Cat# M185-3L; RRID: AB_11123930

**Bacterial and virus strains**		

ECOS Competent *E. coli* DH5α	FUJIFILM	Cat# 310-06236

**Chemicals, peptides, and recombinant proteins**		

Wnt-3A	R&D systems	Cat# 5036-WN-010
Ivermectin	Sigma-Aldrich	I8898; CAS: 70288-86-7
MG132	Sigma-Aldrich	474791; CAS: 133407-82-6
6-bromoindirubin-3’-oxime	Sigma-Aldrich	B1686; CAS: 667463-62-9
Torin2	Selleck	S2817; CAS: 1223001-51-1
Rapamycin	Sigma-Aldrich	R0395; CAS: 53123-88-9
Lactacystin	FUJIFILM Wako Pure Chemical Corporation	333-43681; CAS: 133343-34-7
XAV-939	Tocris Bioscience	3748; CAS: 284028-89-3
FG beads (alkyne beads)	Tamagawa Seiki	TAS8848 N1161
IVM B1a-SNIPER 1	This article	N/A
IVM B1a-SNIPER 2	This article	N/A
IVM B1a-SNIPER 3	This article	N/A
IVM B1a-SNIPER 4	This article	N/A
Lipofectamine RNAiMAX Transfection Reagent	Thermo Fisher Scientific	Cat# 13778030
TransIT-X2 Dynamic Delivery System	Mirus Bio	Cat# MIR6003
TransIT-LT1 Transfection Reagent	Mirus Bio	Cat# MIR2300

**Critical commercial assays**		

Dual-Luciferase Reporter Assay System	Promega	Cat# E1960
Amersham ECL Western Blotting Detection Reagent	GE Healthcare	Cat# RPN2209

**Experimental models: Cell lines**		

Human: HEK293 cells	RIKEN BRC	Cat# RCB1637
Human: DLD-1 cells	ATCC	Cat# CCL-221
Human: HT-29 cells	ATCC	Cat# HTB-38

**Experimental models: Organisms/Strains**		

Zebrafish: wild-type strain RIKEN	The National Bioresource Project of Japan	RIKEN WT (RW)

**Oligonucleotides**		

GeneSolution siRNA Hs_TELO2_1 (siTELO2 #1)	Qiagen	Cat# SI04152904
GeneSolution siRNA Hs_TELO2_3 (siTELO2 #3)	Qiagen	Cat# SI04365249
GeneSolution siRNA Hs_KIAA0683_3 (siTELO2 #4)	Qiagen	Cat# SI00454902
MISSION siRNA Universal Negative Control#1	Merck	Cat# SIC001
Primers for the construction of TELO2 mutant expression vectors, see Data S1	Eurofins Scientific	N/A

**Recombinant DNA**		

Super 8x TOPFlash	Addgene	Cat# 12456 (Veeman et al., 2003)
pRL-SV40	Promega	Cat# E2231
p3xFLAG-CMV10-hTel2	Addgene	Cat# 30214 (Kaizuka et al., 2010)
p3xFLAG-CMV10-hTel2 K749T	This article	N/A
pGEX6p1-TELO2 CTD	This article	N/A
pGEX6p1-TELO2 CTD Δ5	This article	N/A
pGEX6p1-TELO2 CTD Δ6	This article	N/A
pGEX6p1-TELO2 CTD Δ7	This article	N/A
pGEX6p1-TELO2 K749T	This article	N/A
pGEX6p1-TELO2 E753A	This article	N/A
pGEX6p1-TELO2 R759G	This article	N/A
pGEX6p1-TELO2 H761L	This article	N/A
pGEX6p1-TELO2 D763A	This article	N/A
pGEX6p1-TELO2 R767G	This article	N/A

**Software and algorithms**		

ImageJ	National Institutes of Health, USA	ver. 1.52a; RRID:SCR_003070; https://imagej.nih.gov/ij/
BellCurve for Excel	Social Survey Research Information	Ver. 3.22; RRID:SCR_017294; https://bellcurve.jp/ex/

### Resource availability

#### Lead contact

Further information and requests for resources and reagents should be directed to and will be fulfilled by the lead contact Naoyuki Nishiya (nnishiya@iwate-med.ac.jp).

#### Materials availability

Plasmids are available on request. Synthetic routes to all chemical compounds are described using established methodology from commercially available compounds. Antibodies, reagents, zebrafish, and cell lines were obtained from the commercial or academic sources described in the attached key resources table.

### Experimental model and subject details

#### Zebrafish

The zebrafish wild-type (WT) strain RIKEN (the National Bioresource Project of Japan) was used and maintained under a 12-h day/12-h night cycle at 28.5°C. (Nishiya et al., 2014). This study was approved by the Animal Care Ethical Committee of Iwate Medical University, Japan (Permit Number 30–023). Fish care and experimental procedures were in accordance with the Guide for Animal Experimentation of the Animal Care Ethical Committee at Iwate Medical University. Fertilized eggs were obtained by mating adult fish soon after the light was turned on. Embryos were staged according to h postfertilization (hpf) and morphological criteria. Morphologically normal embryos at 5.5–30 hpf were used in the experiments. The fish sex was not determined because the sexual differentiation of zebrafish occurs at 20–30 days postfertilization.

#### Cell lines and culture

HEK293 cells (RIKEN) were cultured in Dulbecco’s Modified Eagle Medium (DMEM) and the human colorectal cancer cell lines DLD-1 and HT-29(ATCC) were cultured in Roswell Park Memorial Institute 1640 (RPMI1640) medium. The cells were supplemented with 2 mM l-glutamine and 10% fetal bovine serum (FBS) in a humidified atmosphere with 5% CO_2_ at 37°C.

### Method details

#### General information for chemical synthesis

To obtain required chemical tools, chemical synthesis was conducted as follows. Unless otherwise noted, the reagents and solvents used here were commercially available and used without further purification. All dry solvents such as MeOH and THF were purchased from Kanto Chemical. For thin-layer chromatography (TLC) analysis, precoated silica gel plates with a fluorescent indicator (60 F_254_, Merck) were used. Flash chromatography was performed with spherical neutral, 0.040–0.050-mm 60 N silica gels (Kanto Chemical) or 60N, 0.040–0.063-mm, 230–400 mesh ASTM silica gels (Merck). Optical rotations were measured using the JASCO P-1010 polarimeter. Melting points (m.p.) were measured using SRS MPA-100 OptiMelt. Infrared (IR) spectra were recorded using the Horiba FT-210 spectrometer. NMR spectra were measured on a JEOL JNM-ECA-500 spectrometer with ^1^H NMR at 500 MHz and ^13^C NMR at 125 MHz. Chemical shifts were reported from the internal solvent peaks for chloroform-*d*_*1*_ (CDCl_3_; ^1^H, δ = 7.26 ppm; ^13^C, δ = 77.16 ppm), dimethylsulfoxide-*d*_*6*_ (DMSO-*d*_*6*_; ^1^H, δ = 2.50 ppm; ^13^C, δ = 39.52 ppm), acetone-*d*_*6*_ [(CD_3_)_2_CO; ^1^H, δ = 2.09 ppm; ^13^C, δ = 205.87 and 30.60 ppm], and methanol-*d*_*4*_ (CD_3_OD; ^1^H, δ = 3.31 ppm; ^13^C, δ = 49.00 ppm). ^1^H NMR data were reported as follows: chemical shift (integration, multiplicity (s = singlet, d = doublet, t = triplet, m = multiplet, br = broad), coupling constants [Hz]). The high-resolution mass spectra (HRMS) were obtained using a JEOL JMS-700 MStation and JEOL JMS-T100LP.

EDCI・HCl : 1-ethyl-3-(3-diemthylaminopropyl)carbodiimide hydrochloride

DMAP: dimethyl-4-aminopyridine

HATU: 1-[bis(dimethylamino)methylene]-1H-1,2,3-triazolo[4,5-b]pyridinium 3-oxide hexafluorophosphate

CuSO_4_・5H_2_O: copper(II) sulfate pentahydrate

DCC: *N,N*’-dicyclohexylcarbodiimide

FmocCl: 9-fluorenylmeethyl chloroformate

[Cu(CH_3_CN)_4_]PF_6_:Tetrakis(acetonitrile)copper(I) hexafluorophosphate

#### Preparation of ivermectin (IVM) B1a linkers

**Scheme 1:**
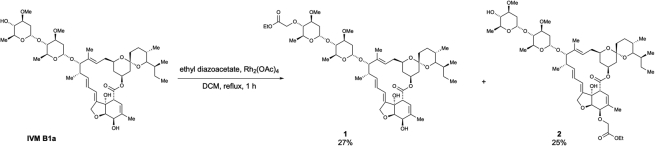
Preparation of 4″-*O*-(ethoxycarbonylmethyl)-ivermectin B1a (1) and 5-*O*-(ethoxycarbonylmethyl)-ivermectin B1a (2)

To a solution of **IVM B1a** (2.0 g, 2.29 mmol) in dichloromethane (45.7 mL, 0.05 M), Rh_2_(OAc)_4_ (10.1 mg, 0.023 mmol, 0.01 equiv.) and ethyl diazoacetate (13% in dichloromethane, 2.0 mL, 2.29 mmol, 1.0 equiv.) were added at room temperature, which was followed by warming of the reaction mixture to 40°C. After stirring for 1 h, the reaction mixture was cooled to room temperature and then concentrated. The crude extract was purified using column chromatography (EtOAc/Hexane = 5/1) to afford 4”-*O*-(ethoxycarbonylmethyl)-ivermectin B1a **(1**) (592.4 mg, 27% yield), according to the method used in a previous study (Nagai et al., 2004), and 5-*O*-(ethoxycarbonylmethyl)-ivermectin B1a (**2**) (549.0 mg, 25% yield) as a white solid (Scheme 1).

2:

m.p.: 106.0–108.0°C.

IR (KBr)νcm^–1^: 3454, 2964, 2931, 2873, 1738, 1450, 1377, 1342, 1300, 1275, 1198, 1173, 1122, 1053, 1011, 985, 933, 903, 874, 835, 756, 665, 633.

^1^H NMR (500 MHz, CDCl_3_)δ(ppm): 5.84-5.82 (m, 1H), 5.73-5.71 (m, 2H), 5.47 (q, *J* = 1.7 Hz, 1H), 5.39 (d, *J* = 3.4 Hz, 1H), 5.34 (ddd, *J* = 16.6, 11.5, 5.2 Hz, 1H), 4.99-4.97 (m, 1H), 4,77 (d, *J* = 3.4 Hz, 1H), 4.68 (dd, *J* = 14.3, 1.7 Hz, 2H), 4.61 (dd, *J* = 14.3, 1.7 Hz, 1H), 4.29 (d, *J* = 5.7 Hz, 1H), 4.24 (d, *J* = 2.3 Hz, 2H), 4.21 (q, *J* = 6.9 Hz, 2H), 4.01 (d, *J* = 5.7 Hz, 1H), 3.93 (brs, 1H), (qd, *J* = 9.7, 6.3 Hz, 1H), 3.76 (qd, *J* = 9.2, 6.3 Hz, 1H), 3.69-3.59 (m, 2H), 3.3.50-3.45 (m, 1H), 3.42 (s, 3H), 3.41 (s, 3H), 3.35 (q, *J* = 2.3 Hz, 1H), 3.23 (t, *J* = 9.2 Hz, 1H), 3.22-3.20 (m, 1H), 3.16 (t, *J* = 9.2 Hz, 1H), 2.53-2.50 (m, 1H), 2.34-2.20 (m, 4H), 1.99-1.95 (m, 1H), 1.89 (brs, 3H), 1.89-1.87 (m, 1H), 1.76-1.73 (m, 1H), 1.66-1.64 (m, 1H), 1.58-1.33 (m, 11 H), 1.49 (s, 3H), 1.28 (t, *J* = 6.9 Hz, 3H), 1.27 (d, *J* = 6.3 Hz, 3H), 1.25 (d, *J* = 6.3 Hz, 3H), 1.17 (d, *J* = 6.9 Hz, 3H), 0.92 (t, *J* = 7.5 Hz, 3H), 0.85 (d, *J* = 6.9 Hz, 3H), 0.82 (d, *J* = 10.0 Hz, 1H), 0.78 (d, *J* = 5.7 Hz, 3H).

^13^C NMR (125)MHz, CDCl_3_)δ(ppm): 173.9, 170.3, 139.6, 137.8 (2C), 135.5, 135.0, 124.8, 120.0, 119.6, 118.3, 98.5, 97.5, 94.8, 81.8, 80.8, 80.4, 79.3, 78.2, 78.0, 76.6, 76.1, 75.4, 68.7, 68.3, 68.1, 67.2, 66.4, 60.8, 56.5, 56.4, 45.7, 41.1, 39.7, 36.9, 35.7, 35.4, 34.5, 34.2, 34.1, 31.2, 28.0, 27.3, 20.2, 19.9, 18.4, 17.7, 17.4, 15.1, 14.2, 12.4, 12.1.

HRMS (EI) *m/z* 983.5344 [M+Na]^+^, calcd 983.5339 for C_52_H_80_NaO_16_^+^.

[α]^21^_D_; +41.4 (c = 0.5, CHCl_3_).

**Scheme 2:**
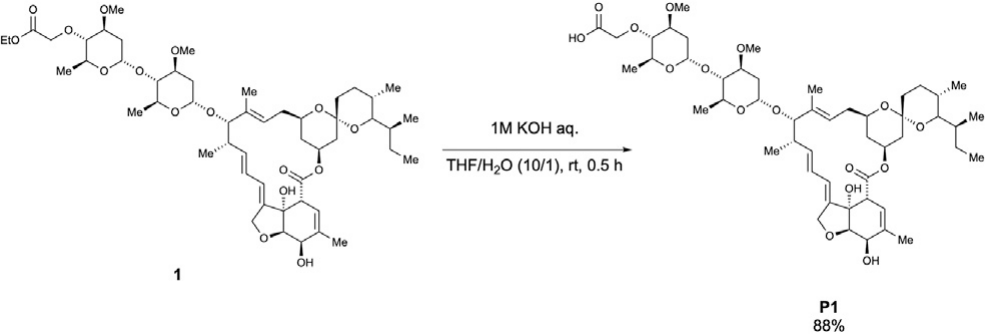
Preparation of 4″-*O*-(carboxymethyl)-ivermectin B1a (P1)

To a solution of **1** (565.3 mg, 0.59 mmol) in THF/H_2_O (22.6 mL, 0.03 M), 1 M KOH solution (2.3 mL, 2.3 mmol, 3.9 equiv.) was added at room temperature. After stirring for 0.5 h, the reaction mixture was quenched with a saturated. NH_4_Cl solution (20 mL) and extracted with CHCl_3_ (15 mL × 3). The combined organic layer was dried over Na_2_SO_4_, filtered, and concentrated. The crude extract was purified using column chromatography (MeOH/CHCl_3_ = 1/9) to afford 4”-*O*-(carboxymethyl)-ivermectin B1a (**P1**) (481.0 mg, 88% yield) (Scheme 2), according to the method used in a previous study (Nagai et al., 2004).

**Scheme 3:**
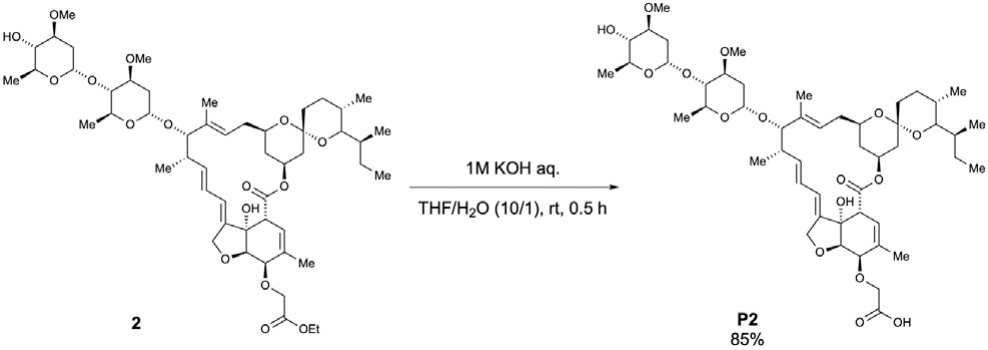
Preparation of 5-*O*-(carboxymethyl)-ivermectin B1a (P2)

According to the method used for the preparation of **P1**, the desired 5-*O*-(carboxymethyl)-ivermectin B1a (**P2)** was obtained as a white solid (569.4 mg, 85% yield) from **2** (687.6 mg, 0.72 mmol) after purification using column chromatography (MeOH/CHCl_3_ =1/9) (Scheme 3).

m.p.: 152.0–154.0°C.

IR (diamond prism)νcm^–1^: 3450, 2964, 2931, 2875, 1736, 1718, 1626, 1452, 1379, 1342, 1300, 1273, 1244, 1198, 1173, 1120, 1074, 1051, 1011, 985, 933, 903, 874, 831, 758, 667, 631.

^1^H NMR (500 MHz, CDCl_3_)δ(ppm): 5.85-5.84 (m, 1H), 5.78-5.67 (m, 2H), 5.50 (brs, 1H), 5.37 (d, *J* = 3.4 Hz, 1H), 5.37-5.29 (m, 1H), 4.9804.96 (m, 1H), 4.76 (d, *J* = 3.4 Hz, 1H), 4.71 (d, *J* = 14.3 Hz, 1H), 4.65 (d, *J* = 14.9 Hz, 1H), 4.38-4.34 (m, 1H), 4.22 (d, *J* = 5.2 Hz, 1H), 4.17-4.14 (m, 1H), 4.02 (d, *J* = 6.3 Hz, 1H), 3.93 (brs, 1H), 3.81 (qd, *J* = 9.2, 6.3 Hz, 1H), 3.75 (qd, *J* = 9.2, 6.3 Hz, 1H), 3.65 (td, *J* = 10.3, 3.4 Hz, 1H), 3.61 (ddd, *J* = 11.5, 8.6, 4.6 Hz, 1H), 3.47 (ddd, *J* = 11.5, 8.6, 4.6 Hz, 1H), 3.41 (s, 3H), 3.40 (s, 3H), 3.30 (brs, 1H), 3.22 (t, *J* = 9.2 Hz, 1H), 3.22-3.19 (m, 1H), 3.15 (t, *J* = 9.2 Hz, 1H), 2.53-2.50 (m, 1H), 2.33-2.24 (m, 3H), 2.20 (dd, *J* = 12.6, 4.6 Hz, 1H), 1.97 (dd, *J* = 12.0, 4.1 Hz, 1H), 1.84 (s, 3H), 1.75-1.72 (m, 1H), 1.65-1.63 (m, 1H), 1.58-1.36 (m, 12H), 1.48 (s, 3H), 1.26 (d, *J* = 6.3 Hz, 3H), 1.24 (d, *J* = 6.3 Hz, 3H), 1.15 (d, *J* = 6.9 Hz, 3H), 0.92 (t, *J* = 6.9 Hz, 3H), 0.84 (d, *J* = 6.3 Hz, 3H), 0.83-0.80 (m, 1H), 0.77 (d, *J* = 5.7 Hz, 3H).

^13^C NMR (125 MHz, CDCl_3_)δ(ppm): 173.5, 172.0, 138.5, 138.4 (2C), 135.0, 134.6, 124.5, 120.7, 120.4, 118.2, 98.4, 97.5, 94.7, 81.7, 80.6, 80.3, 79.3, 78.2, 77.8, 76.6, 75.9, 68.9 (2C), 68.5, 68.1, 67.8, 67.2, 56.4, 56.3, 45.6, 41.1, 39.7, 36.8, 35.7, 35.4, 34.4, 34.2, 34.0, 31.2, 28.0, 27.2, 20.2, 19.9, 18.3, 17.6, 17.4, 15.1, 12.4, 12.0.

HRMS (EI) *m/z* 955.5031 [M+Na]^+^, calcd 955.5026 for C_50_H_76_NaO_16_^+^.

[α]^22^_D_: +26.7 (c = 0.5, CHCl_3_).

**Scheme 4:**
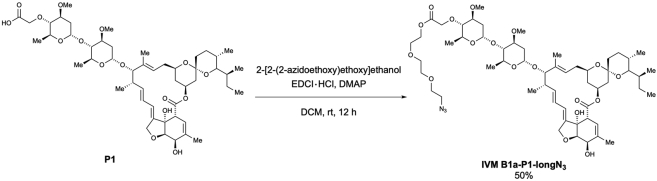
Preparation of 4″-*O*-(azido-PEG3-carbonylmethyl)-ivermectin B1a (IVM B1a-P1-longN_3_)

To a solution of **P1** (38.7 mg, 41.5 μmol) in dichloromethane (0.84 mL, 0.05 M), 2-[2-(2-azidoethoxy)ethoxy]ethanol (249 μL, 124.5 μmol, 3.0 equiv.), DMAP (catalytic amounts), and EDCI・HCl (16.3 mg, 85.0 μmol, 2.0 equiv.) were added at room temperature. After stirring overnight, the reaction mixture was quenched with a saturated. NH_4_Cl solution (1.0 mL) and extracted with CHCl_3_ (1.5 mL × 3). The combined organic layer was dried over Na_2_SO_4_, filtered, and concentrated. The crude extract was purified via preparative TLC (EtOAc/Hexane = 3/1) to afford 4”-*O*-(azido-PEG3-carbonylmethyl)-ivermectin B1a (**IVM B1a-P1-longN**_**3**_) (22.7 mg, 50% yield) as a white solid (Scheme 4).

m.p.: 91.0–93.0°C.

IR (diamond prism)νcm^–1^: 3483, 2964, 2931, 2873, 2104, 1759, 1736, 1712, 1452, 1379, 1342, 1300, 1275, 1244, 1198, 1171, 1122, 1053, 985, 935, 903, 868, 837, 760, 735, 683, 636, 598.

^1^H NMR (500 MHz, CDCl_3_)δ(ppm): 5.86-5.84 (m, 1H), 5.77-5.68 (m, 2H), 5.41 (brs, 1H), 5.34 (ddd, *J* = 16.0, 11.5, 5.2 Hz, 1H), 5.31 (d, *J* = 4.0 Hz, 1H), 4.98-4.96 (m, 1H), 4.76 (d, *J* = 3.4 Hz, 1H), 4.69 (dd, *J* = 14.3, 2.3 Hz, 1H), 4.65 (dd, *J* = 14.3, 2.3 Hz, 1H), 4.44 (d, *J* = 16.0 Hz, 1H), 4.38 (d, *J* = 16.0 Hz, 1H), 4.31-4.28 (m, 3H), 3.96 (d, *J* = 6.3 Hz, 1H), 3.93 (brs, 1H), 3.80 (td, *J* = 9.2 Hz, 6.0 Hz, 2H), 3.73-3.71 (m, 2H), 3.67-3.57 (m, 9H), 3.42 (s, 3H), 3.38 (t, *J* = 5.2 Hz, 2H), 3.35 (s, 3H), 3.28 (q, *J* = 2.3 Hz, 1H), 3.21-3.17 (m, 1H), 3.19 (t, *J* = 9.2 Hz, 1H), 2.95 (t, *J* = 9.2 Hz, 1H), 2.53-2.49 (m, 1H), 2.34-2.19 (m, 4H), 1.98-1.95 (m, 1H), 1.86 (brs, 3H), 1.77-1.74 (m, 1H), 1.66-1.63 (m, 1H), 1.54-1.38 (m, 12H), 1.49 (s, 3H), 1.30 (d, *J* = 6.3 Hz, 3H), 1.23 (d, *J* = 6.3 Hz, 3H), 1.15 (d, *J* = 6.9 Hz, 3H), 0.92 (t, *J* = 7.5 Hz, 3H), 0.85 (d, *J* = 6.9 Hz, 3H), 0.81 (d, *J* = 12.6 Hz, 1H), 0.78 (d, *J* = 5.7 Hz, 3H).

^13^C NMR (125 MHz, CDCl_3_)δ(ppm): 173.8, 170.5, 139.6, 138.1, 137.9, 135.0, 124.7, 120.4, 118.3, 118.0, 98.4, 97.5, 94.7, 84.7, 81.6, 80.8, 80.4, 79.3, 79.1, 78.6, 76.7, 70.6 (C2), 70.1, 69.8, 69.1, 68.6, 68.5, 67.7, 67.2, 67.1 (C2), 63.6, 56.5, 56.3, 50.6, 45.7, 41.1, 39.7, 36.9, 35.7, 35.4, 34.8, 34.4, 34.1, 31.2, 28.0, 27.3, 20.2, 19.9, 18.4, 17.9, 17.4, 15.1, 12.4, 12.1.

HRMS (EI) *m/z* 1112.5882 [M+Na]^+^, calcd 1112.5887 for C_56_H_87_N_3_NaO_18_^+^.

[α]^23^_D_: +26.9 (c = 1.0, CHCl_3_).

**Scheme 5:**
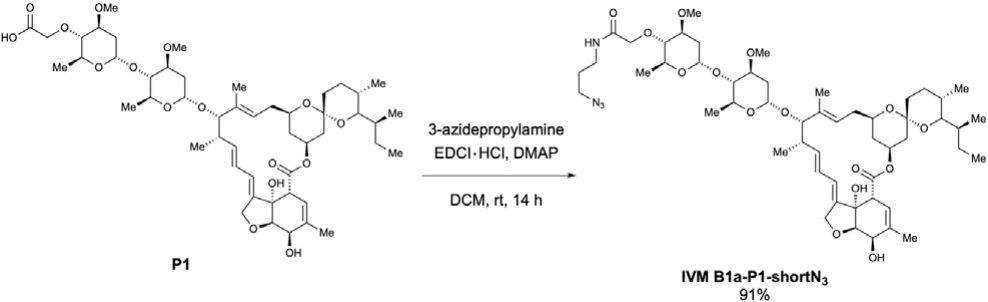
Preparation of 4″-*O*-[2-((3-azidopropyl)amino)-2-oxoethyl]-ivermectin B1a (IVM B1a-P1-shortN_3_)

According to the method used for the preparation of **IVM B1a-P1-longN**_**3**_, the desired 4”-*O*-[2-((3-azidopropyl)amino)-2-oxoethyl]-ivermectin B1a (**IVM B1a-P1-shortN**_**3**_) was obtained as a white solid (44.2 mg, 91% yield) from **P1** (44.7 mg, 47.9 μmol) and 3-azidepropylamine after purification using preparative TLC (MeOH/CHCl_3_ = 1/9) (Scheme 5).

m.p.: 135.8–136.3°C.

IR (diamond prism)νcm^–1^: 2960, 2928, 2876, 2361, 2173, 2096, 1734, 1715, 1666, 1536, 1449, 1376, 1339, 1304, 1266, 1194, 1171, 1119, 1100, 1051, 1008, 982, 930, 900, 868, 753, 677, 664, 598, 546, 535, 520, 508, 499, 483, 472, 459, 445, 419, 403.

^1^H NMR (500 MHz, CDCl_3_)δ(ppm): 7.86 (brt, *J* = 6.3 Hz, 1H), 5.87-5.85 (m, 1H), 5.77-5.68 (m, 2H), 5.42 (brs, 1H), 5.38 (d, *J* = 4.0 Hz, 1H), 5.35 (ddd, *J* = 16.6, 11.5, 5.2 Hz, 1H), 4.99-4.96 (m, 1H), 4.77 (d, *J* = 3.4 Hz, 1H), 4.70 (dd, *J* = 14.3, 2.3 Hz, 1H), 4.65 (dd, *J* = 14.3, 2.3 Hz, 1H), 4,29 (d, *J* = 6.3 Hz, 1H), 4.21 (d, *J* = 16.0 Hz, 1H), 4.09 (d, *J* = 16.0 Hz, 1H), 3.96 (d, *J* = 6.3 Hz, 1H), 3.93 (brs, 1H), 3.83 (dd, *J* = 9.2, 6.3 Hz, 1H), 3.76 (dd, *J* = 9.2, 6.3 Hz, 1H), 3.70-3.58 (m, 3H), 3.44 (s, 3H), 3.43 (s, 3H), 3.42-3.36 (m, 5H), 2.28 (q, *J* = 2.3 Hz, 1H), 3.23-3.20 (m, 2H), 2.93 (t, *J* = 9.2 Hz, 1H), 2.53-2.50 (m, 1H), 2.40 (dd, *J* = 12.6, 5.2 Hz, 1H), 2.35-2.21 (m, 3H), 1.97 (dd, *J* = 12.6, 4.0 Hz, 1H), 1.87 (brs, 3H), 1.85-1.80 (m, 2H), 1.77-1.74 (m, 1H), 1.66-1.64 (m, 1H), 1.58-1.39 (m, 11H), 1.49 (s, 3H), 1.28 (d, *J* = 6.3 Hz, 3H), 1.24 (d, *J* = 6.3 Hz, 3H), 1.16 (d, *J* = 6.9 Hz, 3H), 0.93 (t, *J* = 7.5 Hz, 3H), 0.85 (d, *J* = 6.9 Hz, 3H), 0.82 (d, *J* = 12.0 Hz, 1H), 0.78 (d, *J* = 5.7 Hz, 3H).

^13^C NMR (125 MHz, CDCl_3_)δ(ppm): 174.0, 170.7, 139.8, 138.1 (C2), 135.1, 124.9, 120.5, 118.4, 118.1, 98.2, 97.6, 94.9, 85.7, 81.9, 80.6, 80.5, 79.4, 79.1, 76.8, 71.8, 68.7, 68.6, 68.0, 67.8, 67.3, 67.2, 56.6, 56.3, 49.2, 45.8, 41.2, 39.8, 37.0, 36.3, 35.8, 35.5, 34.8, 34.6, 34.2, 31.3, 28.9, 28.9, 28.1, 27.4, 20.3, 20.1, 18.5, 18.2, 17.5, 15.3, 12.5, 12.2.

HRMS (EI) *m/z* 1037.5674 [M+Na]^+^, calcd 1037.5669 for C_53_H_82_N_4_NaO_15_^+^.

[α]^21^_D_: +49.16 (c = 0.1, CHCl_3_).

**Scheme 6:**
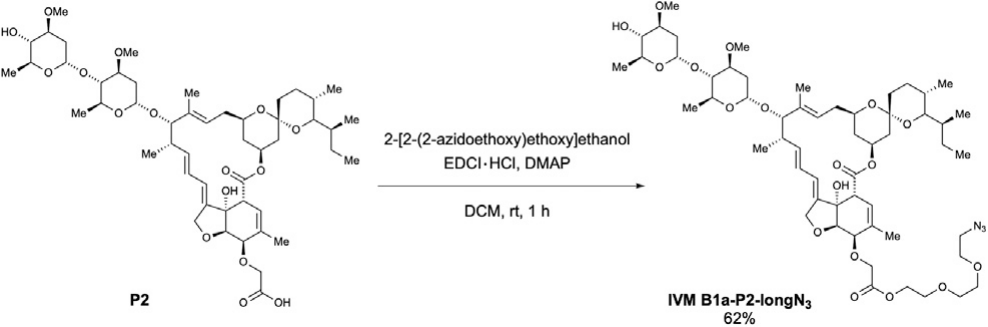
Preparation of 5-*O*-(azido-PEG3-carbonylmethyl)-ivermectin B1a (IVM B1a-P2-longN_3_)

According to the method used for the preparation of **IVM B1a-P1-longN**_**3**_, the desired 5-*O*-(azido-PEG3-carbonylmethyl)-ivermectin B1a (**IVM B1a-P2-longN**_**3**_) was obtained as a white solid (50.9 mg, 62% yield) from **P2** (70.0 mg, 75.0 μmol) after purification using preparative TLC (EtOAc/Hexane = 2/1) (Scheme 6).

m.p.: 85.0–86.0°C.

IR (diamond prism)νcm^–1^: 3458, 2962, 2931, 2873, 2104, 1753, 1736, 1452, 1377, 1342, 1300, 1244, 1198, 1173, 1122, 1051, 1011, 985, 933, 901, 874, 833, 760, 677, 634, 602.

^1^H NMR (500 MHz, CDCl_3_)δ(ppm): 5.83-5.81 (m, 1H), 5.75-5.67 (m, 2H), 5.46 (q, *J* = 1.2 Hz, 1H), 5.37 (d, *J* = 3.4 Hz, 1H), 5.35-5.28 (m, 1H), 4.9804.96 (m, 1H), 4.75 (d, *J* = 3.4 Hz, 1H), 4.67 (dd, *J* = 14.3, 2.3 Hz, 1H), 4.59 (dd, *J* = 14.3, 2.3 Hz, 1H), 4.30-4.27 (m, 3H), 4.27 (s, 2H), 3.99 (d, *J* = 5.7 Hz, 1H), 3.92 (brs, 1H), 3.81 (qd, *J* = 9.7, 6.3 Hz, 1H), 3.75 (qd, *J* = 9.7, 6.3 Hz, 1H), 3.70 (t, *J* = 5.2 Hz, 2H), 3.65 (t, *J* = 5.2 Hz, 2H), 3.63 (s, 6H), 3.62-3.58 (m, 1H), 3.48-3.43 (m, 1H), 3.41 (s, 3H), 3.40 (s, 3H), 3.36 (t, *J* = 5.2 Hz, 2H), 3.32 (q, *J* = 2.3 Hz, 1H), 3.22 (t, *J* = 9.2 Hz, 1H), 3.22-3.19 (m, 1H), 3.14 (t, *J* = 9.2 Hz, 1H), 2.52-2.49 (m, 1H), 2.33-2.18 (m, 4H), 1.98-1.95 (m, 1H), 1.87 (brs, 3H), 1.75-1.72 (m, 1H), 1.65-1.62 (m, 1H), 1.57-1.36 (m, 11H), 1.48 (s, 3H), 1.25 (d, *J* = 6.3 Hz, 3H), 1.24 (d, *J* = 6.3 Hz, 3H), 1.15 (d, *J* = 6.9 Hz, 3H), 0.91 (t, *J* = 6.9 Hz, 3H), 0.83 (d, *J* = 6.3 Hz, 3H), 0.83-0.80 (m, 1H), 0.76 (d, *J* = 5.7 Hz, 3H).

^13^C NMR (125 MHz, CDCl_3_)δ(ppm): 173.8, 170.2, 139.5, 137.8, 135.4, 135.0, 124.7, 120.0, 119.6, 118.2, 98.4, 97.4, 94.7, 81.7, 80.8, 80.4, 79.3, 78.1, 77.9, 76.6, 76.0, 75.4, 70.6, 70.5, 70.0, 69.0, 68.7, 68.3, 68.0, 67.2 (C2), 66.2, 63.7, 56.4, 56.3, 50.6, 45.6, 41.1, 39.6, 36.8, 35.7, 35.4, 34.4, 34.1, 34.0, 31.1, 28.0, 27.2, 20.2, 19.9, 18.3, 17.6, 17.4, 15.1, 12.4, 12.0.

HRMS (EI) *m/z* 1112.5882 [M+Na]^+^, calcd 1112.5877 for C_56_H_87_N_3_NaO_18_^+^.

[α]^22^_D_: +41.4 (c = 1.0, CHCl_3_).

**Scheme 7:**
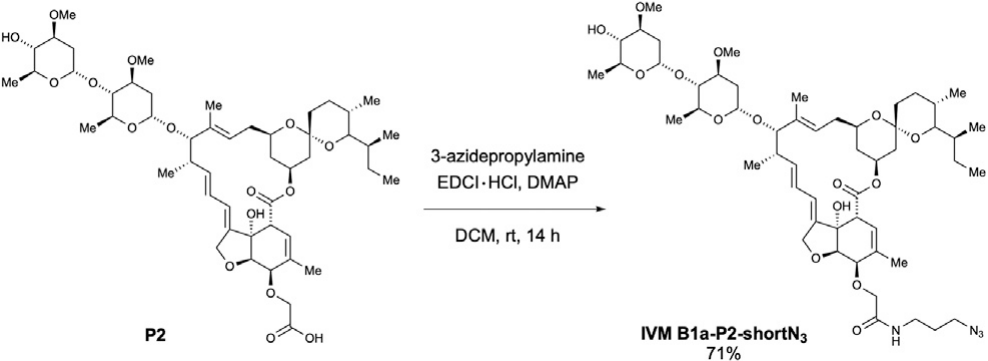
Preparation of 5-*O*-[2-((3-azidopropyl)amino)-2-oxoethyl]-ivermectin B1a (IVM B1a-P2-shortN_3_)

According to the method used for the preparation of **IVM B1a-P1-longN**_**3**_, the desired 5-*O*-[2-((3-azidopropyl)amino)-2-oxoethyl]-ivermectin B1a (**IVM B1a-P2-shortN**_**3**_) was obtained as a white solid (14.5 mg, 71% yield) from **P2** (18.9 mg, 20.3 μmol) after purification using preparative TLC (MeOH/CHCl_3_ = 1/9) (Scheme 7).

m.p.: 136.9–137.9°C.

IR (diamond prism)νcm^–1^: 2960, 2930, 2872, 2096, 1727, 1715, 1667, 1539, 1450, 1379, 1338, 1297, 1268, 1196, 1182, 1168, 1143, 1116, 1106, 1072, 1050, 1010, 983, 931, 903, 874, 833, 756, 667, 605, 597, 583, 547, 530, 516, 500, 485, 470, 453, 437, 422, 412.

^1^H NMR (500 MHz, CDCl_3_)δ(ppm): 7.19 (t, *J* = 5.7 Hz, 1H), 5.85 (td, *J* = 10.3, 2.3 Hz, 1H), 5.79-5.68 (m, 2H), 5.50 (q, *J* = 1.7 Hz, 1H), 5.39 (d, *J* = 3.4 Hz, 1H), 5.39-5.32 (m, 1H), 4.99-4.97 (m, 1H), 4.77 (d, *J* = 3.4 Hz, 1H), 4.71 (dd, *J* = 14.3, 2.3 Hz, 1H), 4.62 (dd, *J* = 14.3, 2.3 Hz, 1H), 4.23-4.11 (m, 4H), 3.99 (d, *J* = 5.7 Hz, 1H), 3.94 (brs, 1H), 3.83 (qd, *J* = 9.2, 6.3 Hz, 1H), 3.77 (qd, *J* = 9.2. 6.3 Hz, 1H), 3.69-3.64 (m, 1H), 3.62 (ddd, *J* = 11.5, 8.6, 5.2 Hz, 1H), 3.50-3.45 (m, 1H), 3.43 (s, 3H), 3.42 (s, 3H), 3.41-3.35 (m, 4H), 3.32 (q, *J* = 2.3 Hz, 1H), 3.24 (t, *J* = 9.2 Hz, 1H), 3.24-3.21 (m, 1H), 3.16 (t, *J* = 9.2 Hz, 1H), 2.54-2.51 (m, 1H), 2.35-2.20 (m, 3H), 1.99-1.96 (m, 1H), 1.84 (brs, 3H), 1.84-1,79 (m, 2H), 1.76-1.73 (m, 1H), 1.67-1.64 (m, 1H), 1.59-1.39 (m, 12H), 1.50 (s, 3H), 1.28 (d, *J* = 6.3 Hz, 3H), 1.25 (d, *J* = 6.3 Hz, 3H), 1.17 (d, *J* = 6.9 Hz, 3H), 0.93 (t, *J* = 7.5 Hz, 3H), 0.85 (d, *J* = 6.9 Hz, 3H), 0.85-0.82 (m, 1H), 0.79 (d, *J* = 5.7 Hz, 3H).

^13^C NMR (125 MHz, CDCl_3_)δ(ppm): 173.7, 170.0, 138.8, 138.4, 135.1, 134.6, 124.6, 120.4, 120.1, 118.3, 98.5, 97.5, 94.8, 81.7, 80.6, 80.3, 79.3, 78.1, 77.8, 77.4, 76.1, 70.0, 68.9, 68.4, 68.1, 67.2 (C2), 56.5, 56.4, 49.1, 45.6, 41.1, 39.7, 36.9, 36.3, 35.7, 35.4, 34.5, 34.1 (C2), 31.2, 29.7, 28.8, 28.0, 27.3, 20.2, 19.9, 18.4, 17.7, 17.4, 15.2, 12.4, 12.1.

HRMS (EI) *m/z* 1037.5674 [M+Na]^+^, calcd 1037.5669 for C_53_H_82_N_4_NaO_15_.

[α]^22^_D_: +46.36 (c = 0.1, CHCl_3_).

#### Preparation of IVM B1a-SNIPERs

**Scheme 8:**
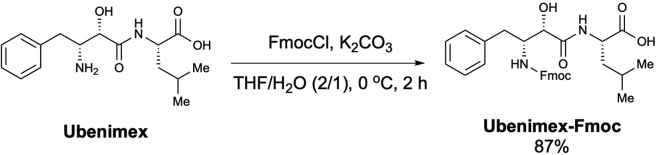
Preparation of *N*-[(2*S*,3*R*)-3-(Fmoc-amino)-2-hydroxy-4-phenylbutyryl]-L-leucine (Ubenimex-Fmoc)

To a solution of ubenimex (15.0 mg, 48.64 μmol) in THF/H_2_O (0.63 mL, 2/1, 0.08 M), FmocCl (12.6 mg, 48.64 μmol, 1.0 equiv.) and K_2_CO_3_ (12.8 mg, 92.9 μmol, 1.9 equiv.) were added at 0 °C. After stirring for 2 h, the reaction mixture was quenched with a 1 N HCl solution (1.0 mL) and extracted with EtOAc (2.0 mL × 3). The combined organic layer was dried over Na_2_SO_4_, filtered, and concentrated to obtain *N*-[(2*S*,3*R*)-3-(Fmoc-amino)-2-hydroxy-4-phenylbutyryl]-L-leucine (**Ubenimex-Fmoc**) as a pale-yellow solid (22.4 mg, 87% yield) (Scheme 8), which was used in subsequent reactions without purification, according to the method used in a previous study (Itoh et al., 2010).

**Scheme 9:**
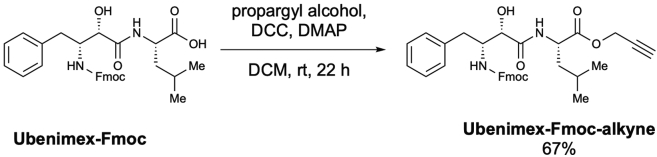
Preparation of *N*-[(2*S*,3*R*)-3-(Fmoc-amino)-2-hydroxy-4-phenylbutyryl]-L-leucine propargyl ester (Ubenimex-Fmoc-alkyne)

To a solution of **Ubenimex-Fmoc** (15.0 mg, 28.27 μmol) in dichloromethane (0.11 mL, 0.25 M), propargyl alcohol (5.0 μL, 84.80 μmol, 3.0 equiv.), DCC (11.6 mg, 56.54 μmol, 2.0 equiv.), and DMAP (0.34 mg, 2.82 μmol, 0.1 equiv.) were added at room temperature. After stirring for 22 h, the reaction mixture was quenched with H_2_O (1.0 mL) and extracted with EtOAc (1.0 mL × 3). The combined organic layer was purified using preparative TLC (EtOAc/Hexane = 2/3) to afford *N*-[(2*S*,3*R*)-3-(Fmoc-amino)-2-hydroxy-4-phenylbutyryl]-L-leucine propargyl ester (**Ubenimex-Fmoc-alkyne**) (10.7 mg, 67% yield) as a colorless oil (Scheme 9).

IR (diamond prism)νcm^–1^: 3301. 2957, 2032, 2359, 2325, 1749, 1706, 1655, 1527, 1503, 1449, 1335, 1272, 1241, 1218, 1150, 1100, 1032, 982, 938, 750, 706, 666, 635, 534, 501.

^1^H NMR (500 MHz, CDCl_3_)δ(ppm): 7.76 (d, *J* = 7.5 Hz, 2H), 7.51 (d, *J* = 7.5 Hz, 1H), 7.48 (d, *J* = 7.5 Hz, 1H), 7.40 (td, *J* = 7.5, 1.7 Hz, 2H), 7.32-7.16 (m, 9H), 5.45-5.44 (m, 1H), 5.15 (brs, 1H), 4.71 (dd, *J* = 15.5, 2.3 Hz, 1H), 4.61 (dd, *J* =15.5, 2.3 Hz, 1H), 4.67-4.63 (m, 1H), 4.38-4.34 (m, 1H), 4.24-4.20 (m, 2H), 4.14 (t, *J* = 6.9 Hz, 2H), 3.06-3.00 (m, 2H), 2.45 (dd, *J* = 2.3, 2.3 Hz, 1H), 1.95-1.92 (m, 1H), 1.72-1.56 (m, 4H), 1.35-1.30 (m, 1H), 1.20-1.08 (m, 1H), 0.87 (d, *J* = 6.3 Hz, 3H), 0.86 (d, *J* = 6.3 Hz, 3H).

^13^C NMR (125 MHz, CDCl_3_)δ(ppm): 172.3, 171.8, 157.5, 143.6, 141.2, 137.7, 129.2, 128.5, 127.8, 127.1, 126.7, 125.1, 125.0, 120.0, 75.4, 73.5, 67.4, 55.7, 52.7, 50.3, 47.0, 40.9, 36.3, 24.8, 22.8, 21.4.

HRMS (EI) *m/z* 591.2471 [M+Na]^+^, calcd 591.2466 for C_34_H_36_N_2_NaO_6_^+^.

[α]^21^_D_: -8.84 (c = 0.5, CHCl_3_).

**Scheme 10:**
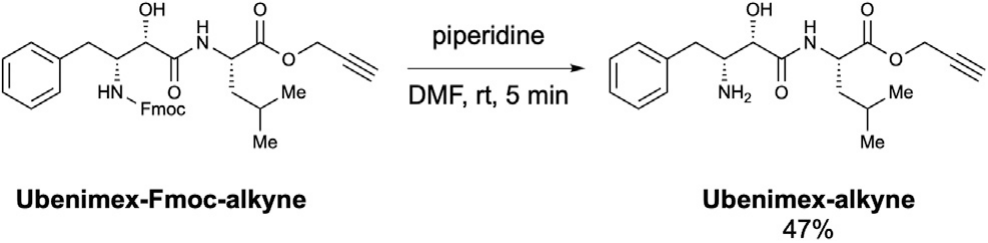
Preparation of *N*-[(2*S*,3*R*)-3amino-2-hydroxy-4-phenylbutyryl]-L-leucine propargyl ester (Ubenimex-alkyne)

To a solution of **ubenimex-Fmoc-alkyne** (6.3 mg, 11.07 μmol) in DMF (0.22 mL, 0.05 M), piperidine (2.2 μmol, 22.15 μmol, 2.0 equiv.) was added at room temperature. After stirring for 5 min, the reaction mixture was concentrated *in vacuo*. The resulting residue was purified using preparative TLC (MeOH/CHCl_3_ = 1/19) to afford *N*-[(2*S*,3*R*)-3amino-2-hydroxy-4-phenylbutyryl]-L-leucine propargyl ester (**Ubenimex-alkyne**) (1.8 mg, 47%) as a colorless oil (Scheme 10).

IR (diamond prism)νcm^–1^: 3307, 3024, 2960, 2926, 2866, 2360, 2334, 1748, 1670, 1521, 1453, 1376, 1275, 1234, 1191, 1150, 1069, 1026, 985, 942, 746, 702, 666, 633, 464, 440, 413.

^1^H NMR (500 MHz, CDCl_3_)δ(ppm): 7.89 (d, *J* = 8.59 Hz, 1H), 7.33-7.30 (m, 2H), 7.25-7.22 (m, 3H), 4.77 (dd, *J* = 16.0, 2.9 Hz, 1H), 4.70 (dd, *J* = 16.0, 2.9 Hz, 1H), 4.67-4.63 (m, 1H), 3.99 (d, *J* = 2.9 Hz, 1H), 3.62-3.59 (m, 1H), 2.99 (dd, *J* = 13.8, 4.6 Hz, 1H), 2.58 (dd, J = 13.8, 10.3 Hz, 1H), 2.47 (dd, *J* = 2.9, 2.3 Hz, 1H), 1.74-1.61 (m, 3H), 0.96 (d, *J* = 6.3 Hz, 3H), 0.94 (d, *J* = 6.3 Hz, 3H).

^13^C NMR (125 MHz, CDCl_3_)δ(ppm): 173.01, 172.0, 129.3, 128.8, 126.8, 75.4, 72.1, 54.4, 52.7, 50.7, 40.8, 37.5, 30.9, 24.9, 22.8, 21.7.

HRMS (EI) *m/z* 347.1971 [M+H]^+^, calcd 347.1965 for C_19_H_27_N_2_O_4_^+^.

[α]^23^_D_: +10.9 (c = 0.1, CHCl_3_).

**Scheme 11:**
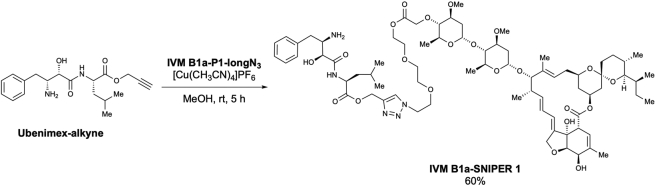
Preparation of ubenimex 4-[1-(4″-*O*-IVM B1a-PEG3-linker)-1*H*-1,2,3-triazolyl]-methyl ester (IVM B1a-SNIPER 1)

To a solution of **IVM B1a-P1-longN**_**3**_ (3.8 mg, 3.49 μmol) in methanol (0.15 mL, 0.02 M), **ubenimex-alkyne** (2.6 mg, 7.51 μmol, 2.2 equiv.) and [Cu(CH_3_CN)_4_]PF_6_ (1.69 mg, 4.53 μmol, 1.3 equiv.) were added at room temperature. After stirring for 5 h, the reaction mixture was concentrated. The resulting residue was purified using preparative TLC (MeOH/CHCl_3_ = 1/10) to afford ubenimex 4-[1-(4”-*O*-IVM B1a-PEG3-linker)-1*H*-1,2,3-triazolyl]-methyl ester (**IVM B1a-SNIPER 1**) (3.0 mg, 60% yield) as a pale-yellow solid (Scheme 11).

m.p.: 94.8–95.8°C.

IR (diamond prism)νcm^–1^: 3347, 3270, 3078, 2957, 2928, 2876, 2360, 2341, 1733, 1683, 1658, 1602, 1540, 1483, 1455, 1426, 1365, 1349, 1325, 1281, 1268, 1238, 1188, 1144, 1119, 1103, 1049, 1015, 984, 868, 816, 797, 745, 692, 672, 608, 593, 521, 473.

^1^H NMR (500 MHz, CDCl_3_)δ(ppm): 7.83-7.81 (m, 1H), 7,77 (s, 1H), 7.33-7.30 (m, 2H), 7.25-7.22 (m, 3H), 5.86 (td, *J* = 10.3, 2.3, Hz, 1H), 5.78-5.69 (m, 2H), 5.43 (brs, 1H), 5.38-5.33 (m, 1H), 5.32 (d, *J* = 5.2 Hz, 1H), 5.32-5.29 (m, 2H), 5.26 (d, *J* = 12.6 Hz, 1H), 4.99-4.97 (m, 1H), 4.77 (d, *J* = 3.4 Hz, 1H), 4.70 (dd, *J* = 14.3, 2.3 Hz, 1H), 4.66 (dd, *J* = 14.3, 2.3 Hz, 1H), 4.63-4.58 (m, 1H), 4.51 (d, *J* = 4.6 Hz, 1H), 4.50 (d, *J* = 4.6 Hz, 1H), 4.43 (d, *J* = 16.0 Hz, 1H), 4.38 (d, *J* = 16.0 Hz, 1H), 4.30-4.29 (m, 3H), 3.97 (d, *J* = 6.3 Hz, 1H), 3.97-3.94 (m, 2H), 3.88-3.78 (m, 4H), 3.68-3.66 (m, 2H), 3,66-3,59 (m, 7H), 3.43 (s, 3H), 3.45 (s, 3H), 3.28 (q, *J* = 2.3 Hz, 1H), 3.22-3.21 (m, 1H), 3.20 (t, *J* = 9.2 Hz, 1H), 2.98-2.95 (m, 1H), 2.96 (t, *J* = 9.2 Hz, 1H), 2.62-2.55 (m, 1H), 2.54-2.50 (m, 1H), 2.34-2.20 (m, 4H), 2.00-1.96 (m, 1H), 1.87 (brs, 3H), 1.78-1.75 (m, 1H), 1,69-1.57 (m, 2H), 1.57-1.40 (m, 11H), 1.50 (s, 3H), 1.30 (d, *J* = 6.3 Hz, 3H), 1.29-1.25 (m, 4H), 1.24 (d, *J* = 6.3 Hz, 3H), 1.16 (d, *J* = 6.9 Hz, 3H), 0.97-0.88 (m, 11H), 0.85 (d, *J* = 6.3 Hz, 3H), 0.83-0.81 (d, *J* = 12.0 Hz, 1H), 0.79 (d, *J* = 5.7 Hz, 3H).

^13^C NMR (125 MHz, CDCl_3_)δ(ppm): 173.8, 173.1, 172.5, 170.5, 142.2, 139.6, 138.2, 138.1, 137.9, 135.0, 129.3, 128.7, 126.7, 124.9, 124.7, 120.4, 118.3, 118.1, 98.4, 97.5, 94.7, 84.7, 81.7, 80.9, 80.4, 79.2, 79.1, 78.6, 72.4, 70.5, 70.4, 69.8, 69.4, 69.0, 68.6, 68.5, 67.7, 67.2, 67.1, 63.5, 58.4, 56.6, 56.3, 54.3, 53.4, 50.7, 50.3, 45.7, 41.2, 40.9, 39.8, 38.0, 36.9, 35.7, 35.4, 34.8, 34.5, 34.1, 31.2, 29.7, 28.1, 27.3, 24.9, 22.9, 21.6, 20.2, 20.0, 18.4, 18.0, 17.4, 15.2, 12.4, 12.1.

HRMS (EI) *m/z* 1458.7775 [M+Na]^+^, calcd 1458.7769 for C_75_H_113_N_5_NaO_22_^+^.

[α]^24^_D_: +25.50 (c = 0.1, CHCl_3_).

**Scheme 12:**
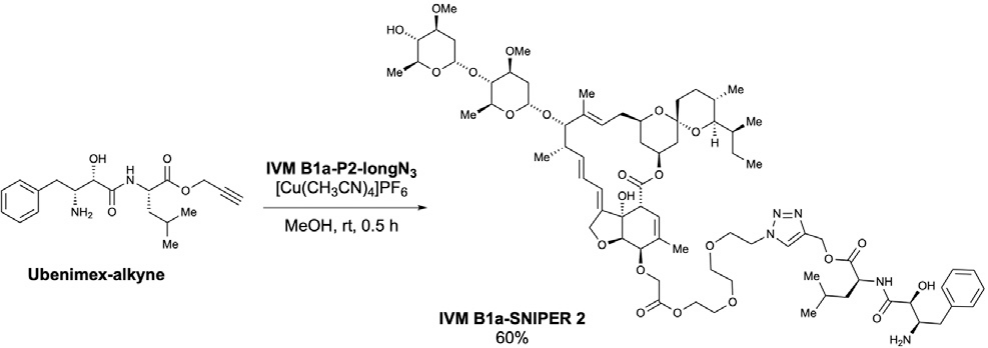
Preparation of ubenimex 4-[1-(5-*O*-IVM B1a-PEG3-linker)-1*H*-1,2,3-triazolyl]-methyl ester (IVM B1a-SNIPER 2)

According to the method used for the preparation of **IVM B1a-SNIPER 1**, the desired ubenimex 4-[1-(5-*O*-IVM B1a-PEG3-linker)-1*H*-1,2,3-triazolyl]-methyl ester (**IVM B1a-SNIPER 2**) was obtained as a pale-yellow solid (7.4 mg, 60% yield) from **IVM B1a-P2-longN**_**3**_ (9.4 mg, 8.62 μmol) after purification using preparative TLC (MeOH/CHCl_3_ = 1/10) (Scheme 12).

m.p.: 94.2–95.3°C.

IR (diamond prism)νcm^–1^: 3624, 2960, 2930, 2866, 2361, 2342, 1995, 1679, 1453, 1389, 1126, 1191, 1177, 1107, 1049, 1106, 984, 871, 800, 585, 507, 475, 450, 420, 411.

^1^H NMR (500 MHz, CDCl_3_)δ(ppm): 7.80-7.78 (m, 2H), 7.33-7.30 (m, 2H), 7.25-7.22 (m, 3H), 5.85-5.83 (m, 1H), 5.77-5.68 (m, 2H), 5.45 (m, 1H), 5.39 (d, *J* = 3.4 Hz, 1H), 5.33-5.29 (m, 1H), 5.30 (d, *J* = 12.6 Hz, 1H), 5.25 (d, *J* = 13.2, Hz, 1H), 5.00-4.98 (m, 1H), 4.78 (d, *J* = 3.4 Hz, 1H), 4.67 (dd, *J* = 14.3, 2.3 Hz, 1H), 4.60 (dd, *J* = 14.3, 1.7 Hz, 1H), 4.59-4.55 (m, 1H), 4.51 (t, *J* = 4.9 Hz, 2H), 4.31-4.28 (m, 6H), 4.07 (d, *J* = 2.9 Hz, 1H), 4.02 (d, *J* = 5.7 Hz, 1H), 3.94 (brs, 1H), 3.84 (t, *J* = 5.2 Hz, 2H), 3.80-3.74 (m, 1H), 3.66 (t, *J* = 4.6 Hz, 2H), 3.65-3.59 (m, 2H), 3.58 (s, 3H), 3.51-3.46 (m, 1H), 3.42 (s, 3H), 3.41 (s, 3H), 3.31 (q, *J* = 2.3 Hz, 1H), 3.24 (t, *J* = 9.2 Hz, 1H), 3.23-3.21 (m, 1H), 3.16 (t, *J* = 9.2 Hz, 1H), 3.02 (dd, *J* = 13.2, 4.6 Hz, 1H), 2.66-2.61 (m, 1H), 2.54-2.51 (m, 1H), 2.35-2.02 (m, 4H), 2.02-1.98 (m, 1H), 1.87 (s, 3H), 1.77-1.74 (m, 1H), 1.69-1.61 (m, 3H), 1.59-1.37 (m, 8H), 1.50 (s, 3H), 1.27 (d, *J* = 5.7 Hz, 3H), 1.25 (d, *J* = 6.3 Hz, 3H), 1.25-1.5 (m, 3H), 1.16 (d, *J* = 6.9 Hz, 3H), 0.94-0.88 (m, 11H), 0.85 (d, *J* = 6.9 Hz, 3H), 0.84-0.82 (m, 1H), 0.78 (d, *J* = 5.7 Hz, 3H).

^13^C NMR (125 MHz, CDCl_3_)δ(ppm): 173.7, 173.0, 172.5, 170.4, 142.2, 139.4, 137.9, 135.3, 135.1, 129.3 (C2), 128.8 (C2), 126.8, 125.0, 124.8, 120.1, 119.7, 118.3, 98.5, 97.5, 94.8, 81.8, 80.8, 80.4, 79.3, 78.2, 77.8, 76.1, 75.4, 71.9, 70.5, 70.4, 69.3, 69.0, 68.8, 68.3, 68.1, 67.2, 66.3, 64.9, 63.7, 58.4, 56.5, 56.4, 54.6, 50.8, 50.3, 45.7, 41.2, 40.7, 39.7, 37.6, 36.8, 35.7, 35.4, 34.5, 34.2, 34.1, 31.2, 29.7, 28.1, 27.3, 24.9, 22.9, 21.6, 20.3, 19.9, 18.4, 17.7, 17.4, 15.1, 12.4, 12.1.

HRMS (EI) *m/z* 1458.7775 [M]^+^, calcd 1458.7769 for C_75_H_113_N_5_NaO_22_^+^.

[α]^24^_D_: +21.28 (c = 0.1, CHCl_3_).

**Scheme 13:**
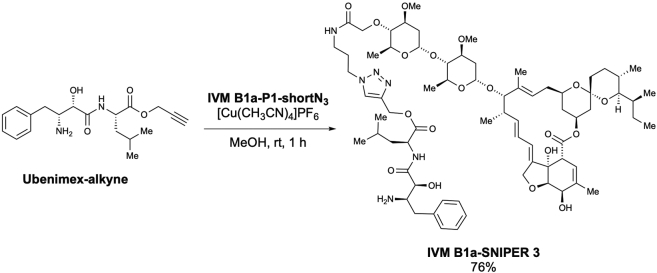
Preparation of ubenimex 4-[1-(4″-*O*-IVM B1a-amide linker)-1*H*-1,2,3-triazolyl]-methyl ester (IVM B1a-SNIPER 3)

According to the method used for the preparation of **IVM B1a-SNIPER 1**, the desired ubenimex 4-[1-(4”-*O*-IVM B1a-amide linker)-1*H*-1,2,3-triazolyl]-methyl ester (**IVM B1a-SNIPER 3**) was obtained as a pale-yellow solid (6.0 mg, 76% yield) from **IVM B1a-P1-shortN**_**3**_ (5.9 mg, 5.77 μmol) after purification using preparative TLC (MeOH/CHCl_3_ = 1/10) (Scheme 13).

m.p.: 113.5–114.9°C.

IR (diamond prism)νcm^–1^: 3351, 3263, 2953, 2925, 2849, 2360, 2334, 1660, 1540, 1456, 1383, 1332, 1263,1173, 1119, 1049, 1012, 983, 898, 844, 747, 696, 666, 599, 557, 457, 424.

^1^H NMR (500 MHz, CDCl_3_)δ(ppm): 8.00 (t, *J* = 6.3 Hz, 1H), 7.77 (s, 1H), 7.66 (d, *J* = 8.0 Hz, 1H), 7.32-7.29 (m, 2H), 7.24-7.22 (m, 3H), 5.86 (td, *J* = 10.3, 2.3 Hz, 1H), 5.77-5.69 (m, 2H), 5.42 (brs, 1H), 5.38 (d, *J* = 3.4 Hz, 1H), 5.38-5.31 (m, 1H), 5.30 (d. *J* = 13.2, Hz, 1H), 5.25 (d, *J* = 12.6 Hz, 1H), 4.99-4.47 (m, 1H), 4.77 (d, *J* = 3.4 Hz, 1H), 4.70 (dd, *J* = 14.3, 2.3 Hz, 1H), 4.65 (dd, *J* = 14.3, 2.3 Hz, 1H), 4.59-4.54 (m, 1H), 4.39 (d, *J* = 6.3 Hz, 1H), 4.37 (d, *J* = 6.9 Hz, 1H), 4.30-4.29 (m, 1H), 4.19 (d, *J* = 16.0 Hz, 1H), 4.07 (d, *J* = 16.0 Hz, 1H), 4.06 (d, *J* = 1.7 Hz, 1H), 3.96 (d, *J* = 5.7 Hz, 1H), 3.94 (brs, 1H), 3.86-3.74 (m, 2H), 3.70-3.59 (m, 4H), 3.43 (s, 6H), 3.29 (q, *J* = 2.3 Hz, 1H), 3.29-3.25 (m, 2H), 3.23-3.20 (m, 1H), 3.21 (t, *J* = 9.2 Hz, 1H), 3.00 (dd, *J* = 13.8, 5.2 Hz, 1H), 2.93 (t, *J* = 9.2 Hz, 1H), 2.67 (m 1H), 2.54-2.50 (m, 1H), 2.40 (dd, *J* = 12.0, 45.2 Hz, 1H), 2.35-2.21 (m, 2H), 2.16-2.10 (m, 2H), 2.00-1.96 (m, 1H), 1.87 (brs, 3H), 1.78-1.74 (m, 1H), 1.68-1.61 (m,4H), 1.57-1.39 (m, 13H), 1.50 (s, 3H), 1.28 (d, *J* = 6.3 Hz, 3H), 1.25-1.24 (m, 3H), 1.24 (d, *J* = 6.3 Hz, 3H), 1.16 (d, *J* = 6.9 Hz, 3H), 0.95-0.89 (m, 10H), 0.85 (d, *J* = 6.9 Hz, 3H), 0.82 (d, *J* = 12.0 Hz, 1H), 0.79 (d, *J* = 5.7 Hz, 3H).

^13^C NMR (125 MHz, CDCl_3_)δ(ppm): 173.8, 173.1, 172.5, 171.4, 142.3, 139.7, 138.0, 137.9, 135.0, 129.3 (C2), 128.8, 128.7, 126.9, 124.7, 124.4, 120.4, 118.3, 118.1, 98.1, 98.0, 97.5, 94.8, 94.7, 85.7, 81.8, 80.6, 80.4, 79.3, 79.1, 71.5, 68.6, 68.5, 67.8, 67.7, 58.6, 56.5, 56.1, 54.8, 51.0, 47.6, 45.7, 41.2, 40.3, 39.7, 37.5, 36.9, 35.7 (C2), 35.4, 34.7, 34.5, 34.1, 31.2, 30.1, 29.7, 28.0 (C2), 27.3, 24.9, 22.8, 21.5, 20.2, 20.0, 18.4, 18.1, 17.4, 15.2, 12.4, 12.1.

HRMS (EI) *m/z* 1361.7748 [M+H]^+^, calcd 1361.7742 for C_72_H_109_N_6_O_19_^+^.

[α]^24^_D_: +11.98 (c = 0.1, CHCl_3_).

**Scheme 14:**
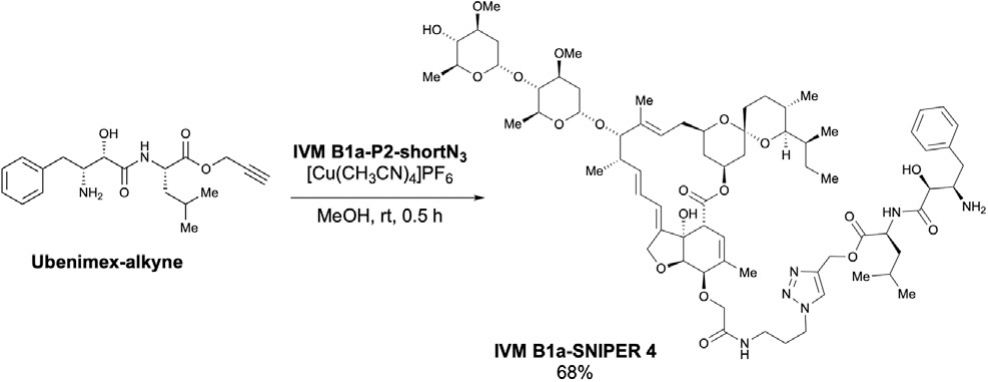
Preparation of ubenimex 4-[1-(5-*O*-IVM B1a-amide linker)-1*H*-1,2,3-triazolyl]-methyl ester (IVM B1a-SNIPER 4)

According to the method used for the preparation of **IVM B1a-SNIPER 1**, the desired ubenimex 4-[1-(5-*O*-IVM B1a-amide linker)-1*H*-1,2,3-triazolyl]-methyl ester (**IVM B1a-SNIPER 4**) was obtained as a pale-yellow solid (7.8 mg, 68% yield) from **IVM B1a-P2-shortN**_**3**_ (8.6 mg, 8.47 μmol) after purification using preparative TLC (MeOH/CHCl_3_ = 1/10) (Scheme 14).

m.p.: 118.2–118.9°C.

IR (diamond prism)νcm^–1^: 3737, 3624, 2960, 2928, 2866, 2361, 2340, 1729, 1667, 1540, 1146, 1383, 1335, 1261, 1184, 1117, 1073, 1046, 898, 861, 752, 703, 605, 487, 446, 418.

^1^H NMR (500 MHz, CDCl_3_)δ(ppm): 8.02-7.96 (m, 1H), 7.80-7.69 (m, 1H), 7.39-7.21 (m, 5H), 5.87-5.85 (m, 1H), 5.79-5.69 (m, 2H), 5.50-5.48 (m, 1H), 5.39-5.22 (m, 3H), 5.27 (d, *J* = 12.6 Hz, 1H), 5.01-4.97 (m, 1H), 4.78 (d, *J* = 2.9 Hz, 1H), 4.73-4.58 (m, 2H), 4.47-4.40 (m, 2H), 4.19-4.11 (m, 2H), 3.94 (brs, 1H), 3.86-3.74 (m, 2H), 3.70-3.60 (m, 2H), 3.51-3.46 (m, 1H), 3.43 (s, 3H), 3.42 (s, 3H), 3.42-3.41 (m, 1H), 3.32-3.27 (m, 1H), 3.26-3.21 (m, 2H), 3.17 (t, *J* = 9.2 Hz, 1H), 2.56-2.50 (m, 1H), 2.36-2.13 (m, 6H), 2.04-1.97 (m, 1H), 1.84 (brs, 3H), 1.78-1.73 (m, 1H), 1.68-1.65 (m, 2H), 1.60-1.39 (m, 15H), 1.50 (s, 3H), 1.28 (d, *J* = 6.3 Hz, 3H), 1.26 (d, *J* = 5.7 Hz, 3H), 1.26-1.25 (m, 6H), 1.17 (d, *J* = 6.9 Hz, 3H), 0.95-0.88 (m, 11H), 0.85 (d, *J* = 6.3 Hz, 3H), 0.85-0.83 (m, 1H), 0.79 (d, *J* = 5.2 Hz, 3H).

^13^C NMR (125 MHz, CDCl_3_)δ(ppm): 173.6, 173.3, 172.5, 170.7, 142.3, 138.7, 138.4, 135.1, 134.4, 129.3 (C2), 128.7 (C2), 126.6, 124.6, 124.1, 120.4, 120.3, 118.2, 98.5, 97.5, 94.8, 81.7, 80.7, 80.3, 79.3, 78.1, 77.9, 76.1, 72.6, 69.7, 68.9, 68.4, 68.1, 67.2 (C2), 58.6, 56.5, 56.4, 54.3, 50.7, 47.6, 45.6, 41.1, 40.9, 39.7, 38.3, 36.9, 35.7 (C2), 35.4, 34.5, 34.1 (C2), 31.2, 30.2, 29.7, 28.0, 27.3, 24.9, 22.9, 21.6, 20.2, 20.0, 18.4, 17.7, 17.4, 15.2, 12.4, 12.1.

HRMS (EI) *m/z* 1383.7567 [M]^+^, calcd 1383.7561 for C_72_H_108_N_6_NaO_19_^+^.

[α]^24^_D_: +21.12 (c = 0.1, CHCl_3_).

#### Plasmid construction

The expression vector encoding human TELO2 K749T was constructed via site-directed mutagenesis using the primers K749Tup and K749Tbot as well as the template p3xFLAG-CMV10-hTel2 (Kaizuka et al., 2010). The expression vector encoding the C-terminal domain (CTD) of human TELO2 was constructed by amplifying the cDNA encoding amino acid residues 584–837 of human TELO2 using primers pGEX6p-BamH1-Tel584 and pGEX6p-XhoI-Tel2term as well as the template p3xFLAG-CMV10-hTel2. The amplified DNA was then cloned into the BamH1/XhoI site of pGEX-6p-1 (Cytiva). The expression vectors encoding the human TELO2 CTD deletion mutants were constructed using primer sets 584–718up and 584–718bot, to introduce a stop codon for TELO2 CTD Δ5, using 584–748up and 584–748bot for TELO2 CTD Δ6, and 584–767up and 584–767bot for TELO2 CTD Δ7. The expression vectors encoding human TELO2 CTD with point mutations were constructed through site-directed mutagenesis using primer sets K749T-ACCup and K749T-ACCbot for TELO2 CTD K749T, E753up and E753bot for TELO2 CTD E753A, R759up and R759bot for TELO2 CTD R759G, H761up and H761bot for TELO2 CTD H761L, D763up and D763bot for TELO2 CTD D763A, and R767up and R767bot for TELO2 CTD R767G. The primer sequences are presented in Data S1. The mutations were verified through sequencing.

#### Wnt/β-catenin pathway inhibitor screening

To identify Wnt/β-catenin pathway inhibitors, five zebrafish embryos per well in round-bottomed 96-well plates were treated with 100 μM test compounds at the 50%-epiboly stage (approximately 5.5 hpf). After a 30-min incubation, the embryos were treated with a GSK3 inhibitor, 6-bromo-indirubin-3′-oxime, at 2 μM and incubated in a humidified box at 28.5°C. The inhibitory activity of the test compounds toward Wnt/β-catenin signaling was screened by evaluating their ability to restore eye development at 30 hpf (Nishiya et al., 2014).

#### Gene knockdown and reconstitution

To perform TELO2 gene knockdown and reconstitute with expression vectors for WT TELO2 or TELO2 K749T, HEK293 cells were transfected with siRNAs using Lipofectamine RNAiMAX (ThermoFisher Scientific) and cultured in 10% FBS/DMEM for 4 days. Cells were replated in 1% FBS/DMEM and transfected with p3xFLAG-CMV10-hTel2 or p3xFLAG-CMV10-hTel2 K749T using TransIT-X2 (Mirus Bio). Transfected cells were cultured for 18 h and used for experiments.

#### Luciferase reporter assay

To evaluate β-catenin/TCF-dependent transcriptional activation, luciferase reporter assays were performed in 6-well plates in the presence of 1% FBS. HEK293 cells were transfected with siRNAs using Lipofectamine RNAiMAX and cultured for 4 days when they were required. Cells were transfected with a β-catenin/TCF-driven firefly luciferase reporter plasmid, Super 8x TOPFlash (Veeman et al., 2003), pRL-SV40 (Promega), which constitutively expressed renilla luciferase under the SV40 promoter, and expression vectors for WT TELO2 or the K749T mutant when they were required, using TransIT-X2. After 24 h of transfection, the cells were treated with ivermectin (IVM, Sigma-Aldrich) and human Wnt3A (R&D systems) for 18 h in 1% FBS/DMEM. The luciferase activity was measured via the Dual Luciferase Assay (Promega). To normalize transfection efficiency, the value obtained from the firefly luciferase activity was divided by that obtained from renilla luciferase activity for each sample. Data from at least three independent experiments were expressed as the means ± standard deviations (SDs).

#### Cell fractionation

To obtain the cytoplasmic proteins, HEK293 or human colorectal cells were treated with compounds for the indicated periods. The cells were washed with ice-cold phosphate buffer saline (PBS), harvested in cell disruption buffer (20 mM HEPES-KOH [pH 7.9], 10 mM KCl, 1 mM MgCl_2_, 0.5 mM DTT, 1 mM NaF, 1 mM Na vanadate, and protease inhibitors), incubated on ice for 15 min, and disrupted by passing through a 27-G needle 10 times. Disrupted cells were centrifuged at 600 × *g* for 5 min at 4°C to remove nuclei, and the supernatant was used as the cytoplasmic fraction.

#### Cell lysis

To obtain protein samples for western blotting, the cells were washed with ice-cold PBS and lysed in radioimmunoprecipitation assay buffer (50 mM Tris-HCl [pH 7.4], 150 mM NaCl, 0.1% sodium dodecyl sulfate [SDS], 1% triton X-100, 0.5% deoxycholate, 1 mM NaF, 1 mM Na vanadate, and protease inhibitors). Disrupted cells were centrifuged at 15,000 rpm for 5 min at 4°C, and the supernatant was used.

#### Western blotting

To detect proteins through western blotting, the following antibodies were used: antibodies against actin and β-catenin (Sigma-Aldrich); GST, TELO2, and TTI1 (Proteintech); Axin2, Cyclin D1, mTOR, ATM, ATR, DNA-PK, phospho-AKT S473, AKT, phospho-p70 S6 kinase T389, and p70 S6 kinase (Cell Signaling Technology); and DDDDK-tag (FLAG-tag; Medical & Biological Laboratories). All antibodies were used at a 1:1,000 dilution, with the exception of TELO2 (1:10,000). In addition, horseradish peroxidase (HRP)-conjugated anti-mouse immunoglobulin G (IgG) or anti-rabbit IgG antibodies were used as secondary antibodies (1:10,000; GE Healthcare), and the Amersham ECL Western Blotting Detection Reagent (GE Healthcare) were used for detection.

#### Affinity chromatography

To identify IVM-binding proteins, IVM B1a-immobilized beads were prepared using FG beads (Alkyne beads, Tamagawa Seiki) and IVM B1a with an azide linker. HEK293 cells were suspended in a cell suspension buffer (20 mM HEPES–NaOH [pH7.5], 150 mM NaCl, 100 mM KCl, 5 μM CaCl_2_, 2.5 mM EGTA, 10% (v/v) glycerol, 1 mM DTT, and protease inhibitors). After a 15-min incubation on ice, 0.5% Tween 20 was added. The suspension was vortexed and then centrifuged at 15,000 rpm for 5 min at 4°C. The supernatant was diluted with the cell suspension buffer to adjust the final concentration of Tween 20 to 0.1%. IVM B1a-immobilized beads were added to the diluted cell lysate, and the mixture was rotated at 4°C for 12 h. Subsequently, the beads were washed with cell suspension buffer containing 0.1% Tween 20. The beads-bound fraction was eluted using the SDS sample buffer (62.4 mM Tris-HCl [pH 6.8], 2% SDS, 10% glycerol, 0.01% bromophenol blue, and 0.1 M DTT). Lysates and bound fractions were analyzed using SDS-PAGE, followed by silver staining or western blotting. Mass spectrometry was performed at Integrale (Naruto, Japan).

#### *In vitro* binding assay

To determine the region that is necessary for binding to ivermectin, glutathione S-transferase (GST)-fusion TELO2 C-terminal domain (CTD) proteins were subjected to *in vitro* binding assays using IVM B1a-immobilized beads. *E. coli* (DH5α, FUJIFILM) expressing GST-fusion TELO2 CTD were suspended in the cell lysis buffer (20 mM HEPES–NaOH [pH 7.5], 150 mM NaCl, 100 mM KCl, 5 μM CaCl_2_, 0.1% Tween 20, 2.5 mM EGTA, 10%(v/v) glycerol, 1 mM DTT, and protease inhibitors) and sonicated on ice. The suspension was centrifuged at 15,000 rpm and 4°C for 5 min. IVM B1a-immobilized beads were added to the *E. coli* lysate, and the mixture was rotated at 4°C for 2 h. The beads were washed thrice with the suspension buffer. The bound fraction was eluted using the SDS sample buffer. Lysates and bound fractions were analyzed using SDS-PAGE, followed by western blotting with an anti-GST-tag antibody.

#### Immunoprecipitation

Coimmunoprecipitation experiments were performed to analyze the effect of short-term ivermectin treatment on the formation of a complex among TELO2 and its binding proteins. HEK293 cells were transfected with expression vectors for WT TELO2 (p3xFLAG-CMV10-hTel2) or TELO2 K749T (p3xFLAG-CMV10-hTel2 K749T) using the TransIT-LT1 Transfection Reagent (Mirus). Cells were treated with 10 μM ivermectin in 1% FBS/DMEM for 1 h, washed with ice-cold PBS, and lysed in TELO2 IP buffer (40 mM HEPES–NaOH [pH7.5], 150 mM NaCl, 2 mM EDTA, 0.3% CHAPS, 10 mM NaF, 1 mM Na Vanadate, and protease inhibitors). FLAG-tagged TELO2 proteins were immunoprecipitated using an anti-DDDDK-tag (FLAG-tag) antibody (Medical & Biological Laboratories) immobilized on protein G Sepharose (Cytiva) at 4°C for 12 h. Bound fractions were washed thrice with TELO2 IP buffer and eluted using the SDS sample buffer. Lysates and bound fractions were analyzed using SDS-PAGE, followed by western blotting.

### Quantification and statistical analyses

Protein levels were quantified using ImageJ software (National Institutes of Health). Data analysis was performed using Excel 2019 (Microsoft) and BellCurve for Excel (Social Survey Research Information Co., Ltd.). The data are presented as the means ± SDs or standard errors of the means. The values of *n* represent the number of experiments. Welch's *t*-test was used for comparing two groups. One-way analysis of variance (ANOVA) with Tukey’s test was used to evaluate statistical significance among more than three groups. A *P*-value of <0.05 indicated statistical significance. ∗*P* < 0.05, ∗∗*P* < 0.01, ∗∗∗*P* < 0.001, n.s.: not significant,

## Data Availability

• The authors declare that all data supporting the findings of this study are available within the article and its supplementary information. All data reported in this paper will be shared by the lead contact upon request. • This article does not report original code. The Mascot analysis data for the IVM B1a-binding proteins are available in the Supplemental Excel spreadsheet (Data S1). • Any additional information required to reanalyze the data reported in this article is available from the lead contact upon request.

## Supplemental information

Document S1. Figures S1–S8 and Table S1

Data S1. IVM B1a-binding proteins, related to Figure 2C
