# Supplementary material for: Ivermectin represses Wnt/β-catenin signaling by binding to TELO2, a regulator of phosphatidylinositol 3-kinase-related kinases
Source: iScience. 2022 Mar 7;25(3):103912. doi: 10.1016/j.isci.2022.103912 (PMC9072907; doi:10.1016/j.isci.2022.103912)
Supplement: Document S1. Figures S1–S8 and Table S1 [file mmc1.pdf]

iScience, Volume *25*

Supplemental information

Ivermectin represses Wnt/β-catenin signaling by binding to TELO2, a regulator of phosphatidylinositol 3-kinase-related kinases

Honami Yonezawa, Akari Ikeda, Ryo Takahashi, Haruka Endo, Yasuyo Sugawara, Mikako Goto, Mirute Kanno, Sosuke Ogawa, Karin Nakamura, Haruki Ujiie, Masato Iwatsuki, Tomoyasu Hirose, Toshiaki Sunazuka, Yoshimasa Uehara, and Naoyuki Nishiya

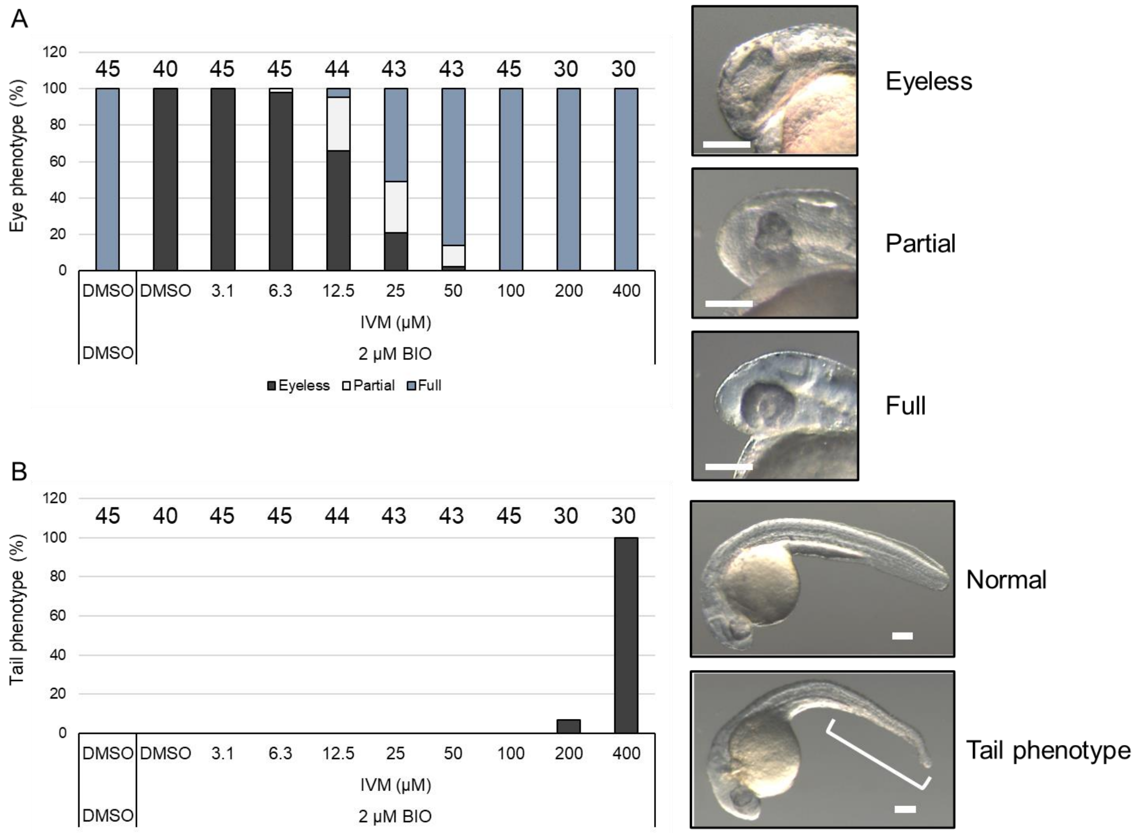

Figure S1. Ivermectin (IVM) suppressed the 6-bromo-indirubin-3′-oxime (BIO)–induced eyeless phenotype in zebrafish embryos, related to Figure 1A. (A and B) IVM restored the eye development in a dose-dependent manner. Zebrafish embryos were pretreated with the indicated concentrations of IVM at 50% epiboly. They were then treated with 2 μM BIO at the shield stage and incubated for 24 h. Images were acquired at 30 hpf. Data are presented as percentages of the total number of embryos treated with the indicated concentrations (left panels). The number of embryos is indicated above the corresponding bars. (A) Eye phenotypes were scored for the eyeless, partially (Partial) or fully (Full) rescued phenotypes. (B) Tail phenotypes were scored. Typical phenotypes are shown on the right. The bracket indicates the tail phenotype. Scale bar, 200 μm.

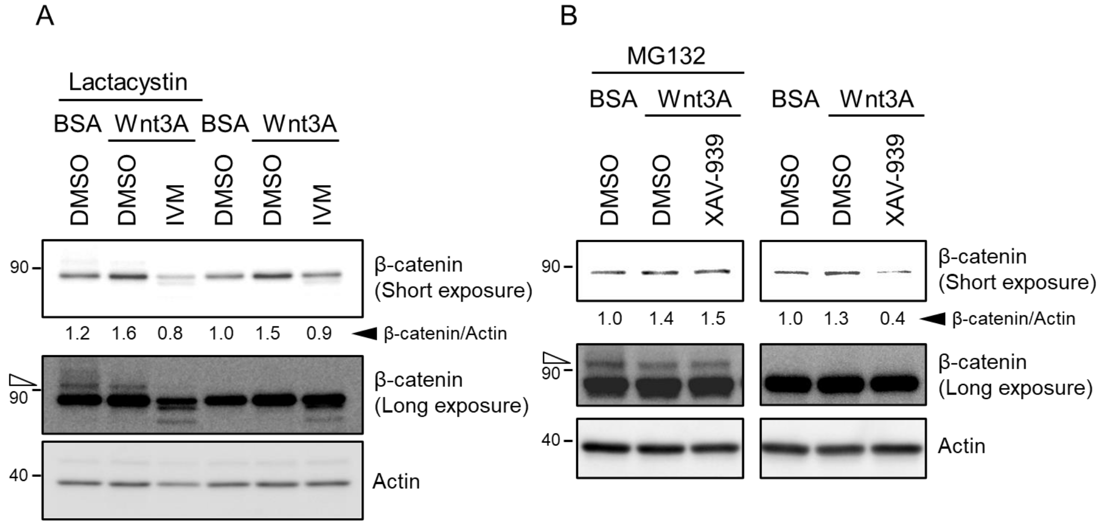

Figure S2. IVM reduced the cytoplasmic β-catenin levels in a proteasome-independent manner, related to Figure 1D. (A and B) Human embryonic kidney (HEK) 293 cells were first treated with 10 μM lactacystin (A) or 25 μM MG132 (B) for 15 min, followed by treatment with 10 μM IVM (A) or XAV939 (B) for 1 h and 50 ng/mL of Wnt3A for 2 h in 1% fetal bovine serum–supplemented Dulbecco's Modified Eagle Medium (FBS/DMEM). The cell lysates were probed with anti-β-catenin and anti-actin antibodies. The open triangles indicate ubiquitinated β-catenin bands. The band intensities were quantified, normalized to the actin levels, and reported as values relative to the control (bovine serum albumin [BSA] and dimethylsulfoxide [DMSO]).

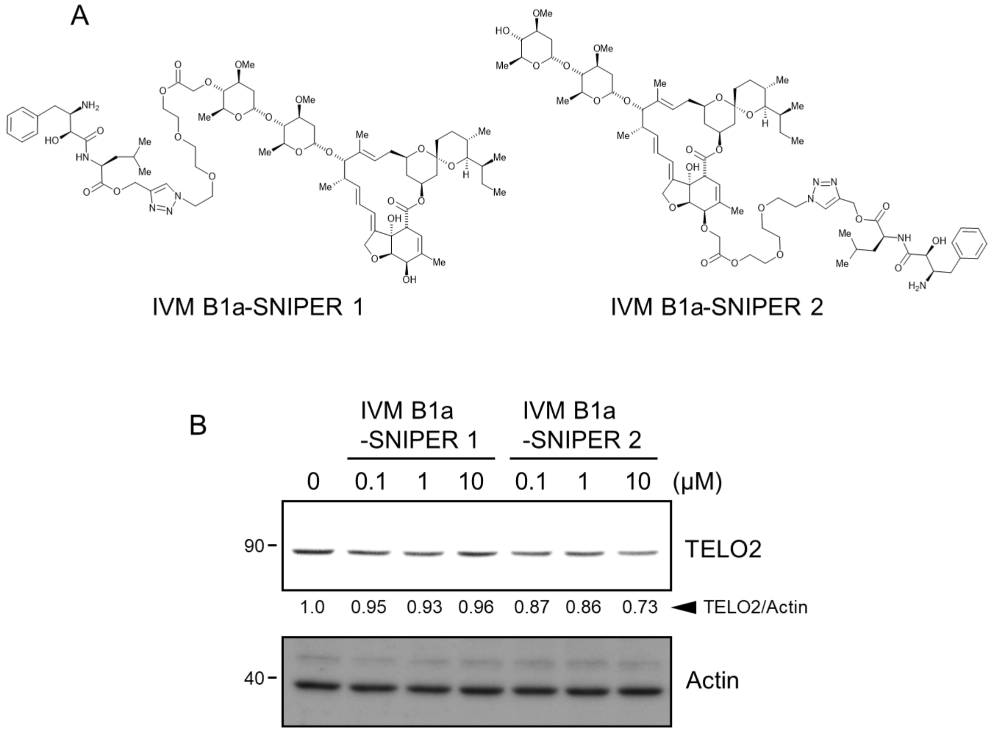

Figure S3. IVM B1a-SNIPERs with a long linker did not reduce the level of telomere length regulation protein 2 homolog (TELO2) , related to Figure 4G. (A) Chemical structures of IVM B1a-SNIPERs 1 and 2. (B) HEK 293 cells were treated with 0.1, 1, or 10 μM IVM B1a-SNIPERs 1 or 2 for 18 h in 1% FBS/DMEM. The cell lysates were probed with anti-TELO2 and anti-actin antibodies. The band intensities were quantified, normalized to the actin levels, and reported as values relative to the control (0 μM IVM B1a-SNIPER).

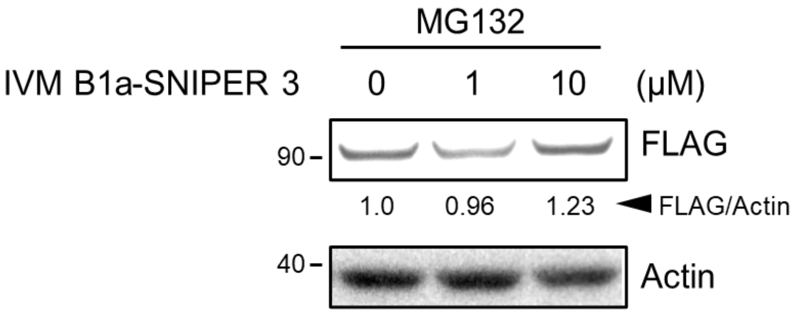

Figure S4. MG132 counteracted the IVM B1a-SNIPER-induced degradation of TELO2, related to Figure 4H. HEK 293 cells were transfected with vectors encoding FLAG-tagged wild-type TELO2 and treated with 1 or 10 μM IVM B1a-SNIPER 3 and 25 μM MG132 for 5 h in 1% FBS/DMEM. The cell lysates were probed with anti-FLAG and anti-actin antibodies. The band intensities were quantified, normalized to the actin levels, and reported as values relative to the control (0 μM IVM B1a-SNIPER 3).

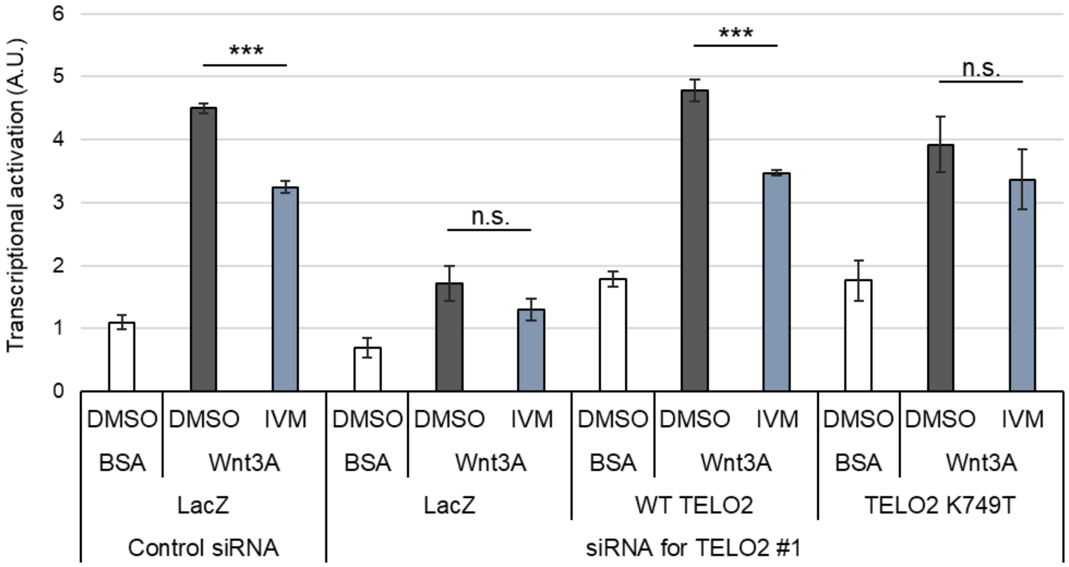

Figure S5. β-catenin/TCF-dependent transcriptional activation in the TELO2 K749T-reconstituted cells is less sensitive to IVM than that observed in wild-type (WT) TELO2-reconstituted cells, related to Figure 5C. HEK 293 cells were transfected with the *TELO2* siRNA for 4 days and cotransfected with an siRNA-resistant FLAG-tagged WT TELO2 or TELO2 K749T expression vector, Super 8x TOPFlash and pRL-SV40. After the cells were treated with 3 μM IVM and 50 ng/mL Wnt3A for 18 h in 1% FBS/DMEM, β-catenin/TCF-dependent transcriptional activation was monitored by a luciferase reporter assay using Super 8x TOPFlash and pRL-SV40. Normalized relative luciferase activities are shown. Data are presented as the means ± standard deviations (SDs; n = 3 biological replicates). ****P* < 0.001, n.s.: not significant, one-way ANOVA with Tukey's test.

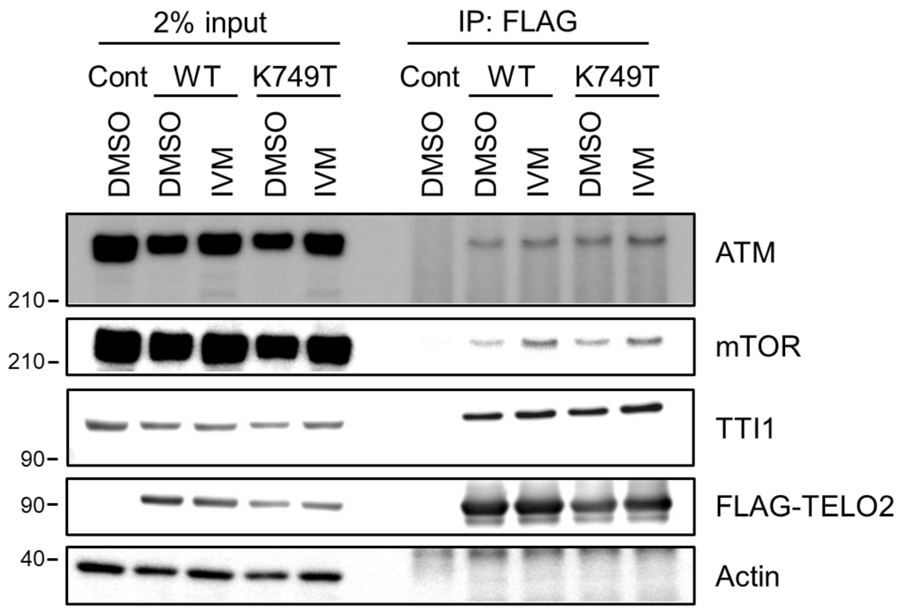

Figure S6. IVM did not affect the formation of a complex among TELO2,TTI1 and phosphatidylinositol 3-kinase-related kinases, related to Figure 6C. HEK 293 cells were transfected with expression vectors for FLAG-tagged wild-type or K749T TELO2, then treated with 10 μM IVM for 1 h in 1% FBS/DMEM. Cell lysates were subjected to immunoprecipitation with an anti-FLAG antibody. The bound fractions were analyzed through western blotting using anti-ATM, anti-mTOR, anti-TTI1, anti-FLAG, and anti-actin antibodies.

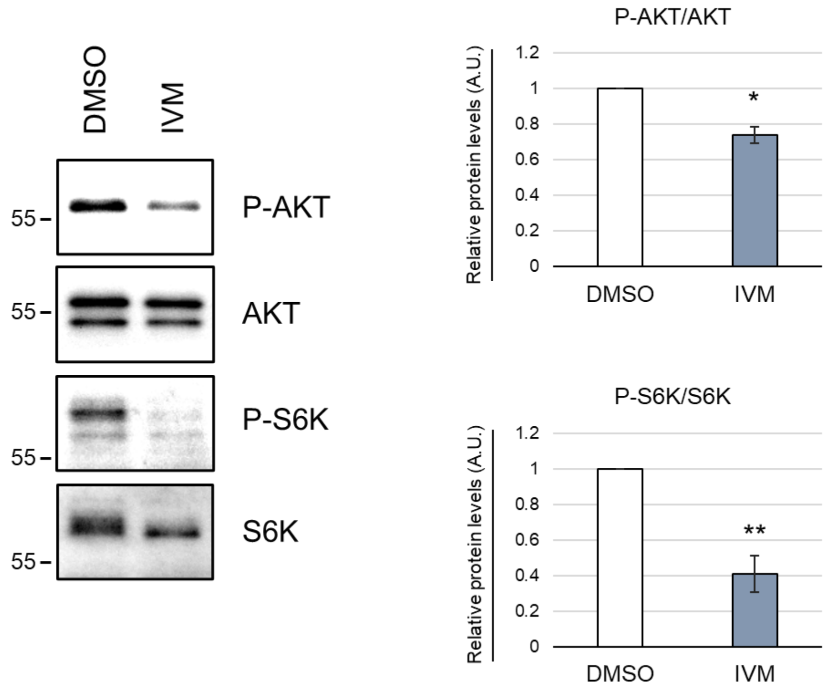

Figure S7. IVM reduced the phosphorylation levels of AKT and S6 kinase in human colorectal adenocarcinoma (HT-29) cells, related to Figure 6C. HT-29 cells were treated with 10 μM IVM for 3 h in 1% fetal bovine serum–supplemented Roswell Park Memorial Institute 1640 Medium. The cell lysates were probed through western blotting with the indicated antibodies (left panels). The band intensities were quantified, normalized to total protein levels, and reported as values relative to the control (DMSO; right panels). The values represent the means ± SDs (n = 3 biological replicates). *$P < 0.05$, **$P < 0.01$, Welch's *t*-test.

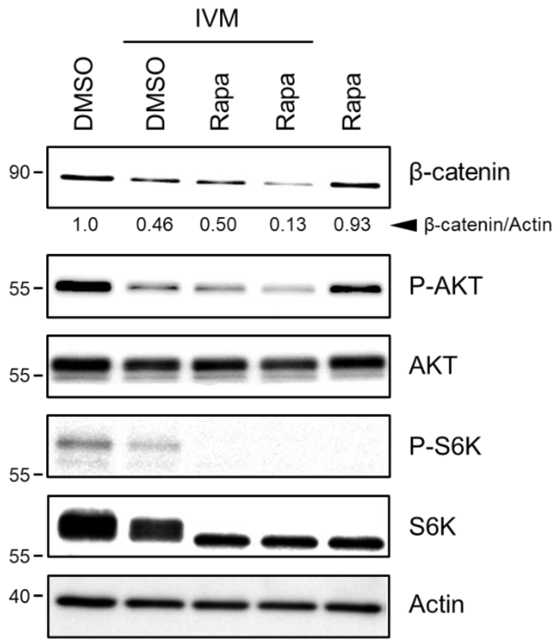

Figure S8. Rapamycin did not preclude the effect of ivermectin (IVM) , related to Figure 6D. HEK 293 cells were first treated with 0.1 μM rapamycin (Rapa) for 15 min, followed by treatment with 10 μM IVM for 3 h in 1% FBS/DMEM. The cell lysates were probed through western blotting with the indicated antibodies. The band intensities were quantified, normalized to the actin levels, and reported as values relative to the control (DMSO).

Table S1. Primers for the construction of TELO2 mutant expression vectors, related to STAR Methods

Primer name	Sequence
K749Tup	5’-GCTGTGGCCATGGGCACGGCCCTGCTGGAATTC-3’
K749Tbot	5’-GAATTCCAGCAGGGCCGTGCCCATGGCCACAGC-3’
pGEX6p-BamH1-Tel584	5’-CATGGATCCACGGTCACAGACCCGGCCCCG-3’
pGEX6p-XhoI-Tel2term	5’-CATGCTCGAGCTAGGGAGACGCGGGTGGGAG-3
584-718up	5’-CTTCGACCTCTTGGGATAAGACCAGCTGGTTC-3’
584-718bot	5’-GAACCAGCTGGTCTTATCCCAAGAGGTCGAAG-3’
584-748up	5’-GCTGTGGCCATGGGCTAGGCCCTGCTGGAATTC-3’
584-748bot	5’-GAATTCCAGCAGGGCCTAGCCCATGGCCACAGC-3’
584-767up	5’-GATGCCTACGTGCGCTAGGGGCTGTTGTCG-3’
584-767bot	5’-CGACAACAGCCCCTAGCGCACGTAGGCATC-3’
K749T-ACCup	5’-GCTGTGGCCATGGGCACCGCCCTGCTGGAATTC-3’
K749T-ACCbot	5’-GAATTCCAGCAGGGCGGTGCCCATGGCCACAGC-3’
E753up	5’-GGCAAGGCCCTGCTGGCATTCGTGTGGGCC-3’
E753bot	5’-GGCCCACACGAATGCCAGCAGGGCCTTGCC-3’
R759up	5’-GTGTGGGCCCTTGGCTTCCACATCGATG-3’
R759bot	5’-CATCGATGTGGAAGCCAAGGGCCCACAC-3’
H761up	5’-GCCCTTCGCTTCCTCATCGATGCCTAC-3’
H761bot	5’-GTAGGCATCGATGAGGAAGCGAAGGGC-3’
D763up	5’-CTTCGCTTCCACATCGCTGCCTACGTGCGC-3’
D763bot	5’-GCGCACGTAGGCAGCGATGTGGAAGCGAAG-3’
R767up	5’-ATCGATGCCTACGTGGGCCAGGGGCTGTTGTCG-3’
R767bot	5-‘CGACAACAGCCCCTGGCCCACGTAGGCATCGAT-3’
